# M2 microglia-derived extracellular vesicles promote white matter repair and functional recovery via miR-23a-5p after cerebral ischemia in mice

**DOI:** 10.7150/thno.68895

**Published:** 2022-04-24

**Authors:** Yongfang Li, Ze Liu, Yaying Song, Jia-ji Pan, Yixu Jiang, Xiaojing Shi, Chang Liu, Yuanyuan Ma, Longlong Luo, Muyassar Mamtilahun, Zhiyu Shi, Haroon Khan, Qing Xie, Yongting Wang, Yaohui Tang, Zhijun Zhang, Guo-Yuan Yang

**Affiliations:** 1 Department of Rehabilitation Medicine, Ruijin Hospital, School of Medicine, Med-X Research Institute and School of Biomedical Engineering, Shanghai Jiao Tong University, Shanghai 200025, China.; 2 Department of Neurology, Renji Hospital, School of Medicine, Shanghai Jiao Tong University, Shanghai 200127, China.; 3 Department of Neurosurgery and Neurology, Huashan Hospital, Shanghai Medical College, Fudan University, Shanghai Clinical Medical Center of Neurosurgery, Shanghai Key Laboratory of Brain Function and Restoration and Neural Regeneration, Shanghai, 200032, China.; 4 Department of Critical Care Medicine and Neurosurgery of Huashan Hospital, State Key Laboratory of Medical Neurobiology, MOE Frontiers Center for Brain Science, and Institutes of Brain Science, Fudan University, Shanghai, 200032, China.

**Keywords:** extracellular vesicles, ischemia, microglia, oligodendrocyte precursor cells, white matter

## Abstract

**Rationale:** White matter repair is critical for the cognitive and neurological functional recovery after ischemic stroke. M2 microglia are well-documented to enhance remyelination and their extracellular vesicles (EVs) mediate cellular function after brain injury. However, whether M2 microglia-derived EVs could promote white matter repair after cerebral ischemia and its underlying mechanism are largely unknown.

**Methods:** EVs were isolated from IL-4 treated microglia (M2-EVs) and untreated microglia (M0-EVs). Adult ICR mice subjected to 90-minute transient middle cerebral artery occlusion received intravenous EVs treatment for seven consecutive days. Brain atrophy volume, neurobehavioral tests were examined within 28 days following ischemia. Immunohistochemistry, myelin transmission electron microscope and compound action potential measurement were performed to assess white matter structural remodeling, functional repair and oligodendrogenesis. The effects of M2-EVs on oligodendrocyte precursor cells (OPCs) were also examined *in vitro*. EVs' miRNA sequencing, specific miR-23a-5p knockdown in M2-EVs and luciferase reporter assay were used to explore the underlying mechanism.

**Results:** M2-EVs reduced brain atrophy volume, promoted functional recovery, oligodendrogenesis and white matter repair *in vivo*, increased OPC proliferation, survival and differentiation* in vitro*. miR-23a-5p was enriched in M2-EVs and could promote OPC proliferation, survival and maturation, while knocking down miR-23a-5p in M2-EVs reversed the beneficial effects of M2-EVs both *in vitro* and *in vivo*. Luciferase reporter assay showed that miR-23a-5p directly targeted Olig3.

**Conclusion:** Our results demonstrated that M2 microglia could communicate to OPCs through M2-EVs and promote white matter repair via miR-23a-5p possibly by directly targeting Olig3 after ischemic stroke, suggesting M2-EVs is a novel and promising therapeutic strategy for white matter repair in stroke and demyelinating disease.

## Introduction

Stroke is a life threatening disease globally with high morbidity, mortality, healthcare costs and limited therapies 1, 2. Ischemic stroke, accounting for more than 80% of stroke, causes not only gray matter but also remarkable white matter injury, since white matter is more vulnerable to ischemia than gray matter 3-5. The loss of myelin and white matter integrity leads to impulse conduction disorder and failure of metabolic and trophic support to axons, resulting in axon degeneration as well as profound cognitive impairment and sensorimotor dysfunction in clinic 3, 6. Increasing evidence suggests that white matter repair after stroke is closely related to neurological functional recovery 7-9. However, enhancing white matter remodeling and repair after stroke is often neglected 4. Therefore, promoting white matter remodeling and repair could be a novel therapeutic target for cognitive and neurological functional recovery after stroke.

In ischemic stroke, white matter injury occurs along with neuronal death, blood-brain barrier (BBB) damage and other pathological changes, which is caused by the death of mature myelinating oligodendrocytes and oligodendrocyte precursor cells (OPCs), in addition to the loss of myelin and degeneration of axons 3, 6, 10. The remodeling and repair of white matter injury mainly relies on the activation, proliferation, migration and differentiation of OPCs into mature oligodendrocytes and wrapping axons to form myelin 10, 11. However, these processes, especially the differentiation of OPCs, are limited and insufficient to support white matter repair under ischemic context 4, 12, 13. Approaches to improve OPC differentiation could be beneficial for cognitive and neurological recovery after stroke.

Microglia are resident immune cells and can polarize to either pro-inflammatory M1 phenotype or anti-inflammatory/pro-regenerative M2 phenotype in different phases of stroke 14, 15. In ischemic stroke, endogenous oligodendrogenesis was closely associated with the dynamic transition of microglia from M1 to M2 phenotype 16, while improved cognitive function after IL-4 administration was related to M2 microglia polarization 17, suggesting that M2 microglia could promote white matter remodeling and repair after stroke. Indeed, M2 microglia were proved to facilitate white matter repair through phagocytosing myelin debris 18, 19 and producing regenerative factors such as insulin-like growth factor 1, transforming growth factor α (TGF-α) and galectin-3, activin-A in the development and demyelinating disease 20-23. Thus, M2 microglia could be used to promote white matter remodeling and repair.

Extracellular vesicles (EVs) are secreted by cells with a diameter ranging in 30-150 nm and could easily cross the BBB 24. Growing evidence revealed that EVs could be a replacement for cell therapy to exert restorative therapeutic potentials via delivering EVs' miRNAs, mRNAs, proteins and metabolites to different brain cells after stroke 25-27. Previous studies have highlighted the effects of mesenchymal stem cell-derived EVs and serum EVs in promoting remyelination 28, 29. Recently, M2 microglia-derived EVs (M2-EVs) were proved to promote OPC migration and maturation as well as remyelination in lysolecithin-induced myelin injury 30. However, the therapeutic effect of M2-EVs on white matter remodeling and repair after ischemic stroke and its underlying mechanism are largely unknown.

MicroRNAs (miRNAs) are small non-coding RNA molecules that post-transcriptionally regulate gene expression. Recent researches reveal that miRNAs are critically important in modulating the proliferation, survival or differentiation of OPCs and white matter repair 31-33. miR-146a was proved to increase myelin expression and decrease OPC apoptosis via inhibiting its target gene IRAK1 after ischemic stroke in rat 34. miR-219 promoted OPC differentiation and myelination through directly repressing Sox6, PDGFRα and Lingo1 expression in the development 35, 36. MSC-derived EVs' miR-134 could protect oligodendrocyte against ischemia-induced apoptosis by targeting caspase-8 expression 37. Intravenously administered miR-17-92 cluster-enriched MSC EVs further enhanced oligodendrogenesis, neurogenesis and functional recovery after ischemic stroke, possibly via targeting phosphatase and tensin homolog (PTEN) and activating Akt signaling 38, 39. miR-23 was one of the most abundant miRNAs in oligodendrocyte, and miR-23a was proved to promote OPC differentiation via targeting PTEN10, laminB1 and fine tuning the activities of Akt/mTOR and mitogen-associated protein kinase pathway in myelin development 40, 41. Recent study revealed that regenerative microglial EVs could restore the functions of protective microglia/macrophages and promoted OPC differentiation via EV-carried transmembrane protein, TNF, after ischemic stroke 42. However, whether miRNAs in M2-EVs could modulate the functions of OPCs remains unknown.

In this study, we investigated the therapeutic effect and mechanism of systemically administrated M2-EVs on white matter remodeling and repair after transient middle cerebral artery occlusion (tMCAO) in mice. Our results demonstrated that M2-EVs treatment promoted oligodendrogenesis, white matter repair and functional recovery via M2-EVs' miR-23a-5p after cerebral ischemia. Further mechanism study showed that miR-23a-5p, miR-221-3p, miR129-5p and miR-155-5p contained in M2-EVs promoted the survival and differentiation of OPCs, especially M2-EVs' miR-23a-5p could promote OPC differentiation possibly via directly targeting Olig3.

## Materials and Methods

### M2 microglia stimulation and identification

Microglial BV2 cells were cultured in Dulbecco's modified Eagle medium (DMEM, HyClone, Logan, UT) supplemented with 10% heat-inactivated Fetal Bovine Serum (FBS, Gibco) and 1% penicillin streptomycin antibiotic (Meilunbio, Dalian, CN). After seeding cells at 1.5×10^6^ in 10 cm dishes for 6 hours, they were treated with 20 ng/mL recombinant mouse interleukin 4 (IL-4, Novoprotein, Shanghai, CN) for 48 hours to polarize them to M2 phenotype 21, 43. M2 microglia were further identified using quantitative real-time polymerase chain reaction (qRT-PCR), western blot analysis and immunostaining.

### Isolation, identification and labeling of microglial EVs

Microglia-derived EVs were extracted from untreated microglia culture supernatant (M0-EVs) and IL-4-treated microglia culture supernatant (M2-EVs) using serial, sequential centrifugation and ultracentrifugation as previously described 43. To avoid the contamination of EVs in FBS, FBS was ultracentrifuged at 100,000 *g* for 16hours at 4 °C using a SW32Ti rotor (Optima Ultracentrifuge, Beckman Coulter Life Sciences, GER). After seeding for 6 hours, microglia were washed by PBS and cultured for 48 hours with or without IL-4 treatment in EVs-depleted medium. Then the microglia cell supernatant was collected and went through differential ultracentrifugation at 300 *g* for 10 minutes, 2000 *g* for 10 minutes, 10,000 *g* for 30 minutes at 4 °C to deplete cells and debris, and 100,000 *g* for 70 minutes at 4 °C to pellet EVs. The obtained microglial EVs were further purified by washing once with PBS at 100,000 *g* for 70 minutes at 4°C and suspended in PBS for characterization and utilization.

The protein concentration of microglial EVs was examined by BCA protein assay (Thermo Scientific, Waltham, MA). The isolated microglial EVs were detected for the EVs' marker CD63, tumor of susceptibility gene 101 (TSG 101), and endoplasmic reticulum marker, calnexin, using western blot. To characterize the morphology of every single EVs, EVs were stained with 1% uranyl acetate and detected by transmission electron microscope (TEM, Thermo Scientific, Waltham, MA). The particle number and size distribution of microglial EVs were evaluated by Nanoparticle tracking analysis (NTA, Brookhaven, NY) according to the device instruction.

For microglial EVs uptake experiments, EVs were labeled with red fluorescent dye PKH26 (Sigma-Aldrich, MO) according to the kit instruction with minor modification. Briefly, 2 μL PKH26 dye and EVs were respectively dissolved in 500 μL Diluent C, then mixed together and incubated for 5 minutes at room temperature (RT). To stop excess labelling, 200 μL EVs-depleted FBS was added. The labeled microglial EVs were washed in PBS at 100,000 *g* for 70 minutes at 4 °C and suspended in 100 μL PBS for further use.

### Animal Experimental design

All animal procedures were performed according to the Institutional Animal Care and Use Committee (IACUC) of Shanghai Jiao Tong University, Shanghai, China (Permission number: Bioethics 2012022). The study was followed by ARRIVE (Animal Research: Reporting *in vivo* Experiments) guideline with careful attention and appropriate managements to minimize the discomfort and pain of the animals. The experimental design was depicted in Figure **2A**. A total of 122 ICR male mice aged 8-10 weeks (JSJ, Shanghai, CN) were used in this study. Mice were subjected to 90-minute tMCAO, and received systemic administration of 200 μL PBS or EVs (100 μg per mouse) for 7 consecutive days (1 to 7 days after tMCAO, once daily) via tail vein. Brain atrophy volume, neurological score, rotarod test, hanging wire test, corner test, step-through and T-maze were examined within 28 days following tMCAO. The structural remodeling and electrophysiological function of white matter were evaluated 28 days after tMCAO using myelin immunostaining, TEM and compound action potential (CAP) measurement.

### Transient middle cerebral artery occlusion (tMCAO) in mice

The procedure of tMCAO was performed as previously described 44. Briefly, animals were smoothly anesthetized with 1.5% isoflurane (RWD, Shenzhen, CN) inhalation. Carotid arteries were gently separated, a 6-0 silicone-coated suture (Covidien, MN) was carefully inserted into the external carotid artery and then reversely advanced into internal carotid artery until reaching the origin of the middle cerebral artery. After 90 minutes, the reperfusion was achieved through withdrawing the suture. A laser Doppler flowmetry (Moor Instruments, Devon, UK) and laser speckle (RWD, Shenzhen, CN) was used to detect the blood flow of the middle cerebral artery to monitor the success of occlusion and reperfusion. Occlusion was determined by the blood flow lower than 20% of baseline and reperfusion by blood flow higher than 80% of baseline. Sham group mice underwent the same operation except for the suture insertion and occlusion.

### Brain infarct volume assessment

Mice were sacrificed at 14 and 28 days after tMCAO, and brain atrophy volume was examined as described before 45. Briefly, brain tissues were sequentially immersed in 4% paraformaldehyde (PFA, Sinopharm Chemical Reagent, Shanghai, CN) and 30% sugar solution at 4°C after cardiac perfusion of PBS and 4% PFA. The mouse brain was cut into 30 μm coronal sections from the anterior commissure to the hippocampus. Brain slices (300 μm apart) were stained with 0.05% Cresyl violet acetate (Sigma-Aldrich), the area of the contralateral and the ipsilateral hemisphere was measured using Image *J* software (NIH, Bethesda, MD). The atrophy area denoted as ∆S was calculated by subtracting ipsilateral area from contralateral area. The thickness between two adjacent brain slices was described as height (H), and the atrophy area of two adjacent slices was denoted as ∆Sn and ∆Sn+1. Total brain atrophy volume was then calculated according to the formula: V= ∑H/3×[∆Sn+(∆Sn×∆Sn+1)^1/2^+∆Sn+1].

### Neurobehavioral and cognitive tests

Neurobehavioral tests including modified neurological severity score (mNSS), rotarod test, hanging wire and corner test were performed before and at 1, 3, 7, 14, 21 and 28 days after tMCAO 46-48. The cognitive tests including T-maze spontaneous alternation and step-through passive avoidance test were performed at 14 and 28 days after tMCAO 47, 49. All behavioral tests were carried out by an investigator blinded to the experimental treatment. The detailed protocols are described in the supplemental materials.

### TEM of myelin

Mice were transcardially perfused with a mixture of 2% PFA and 2.5% glutaraldehyde, then the striatum was roughly separated and post-fixed with 2.5% glutaraldehyde overnight at 4 °C. The peri-infarct region of the striatum was further dissected and cut into 2 mm × 1 mm × 1 mm cuboid to distinguish the direction of myelin fibers under a stereoscope (Leica, GER). Samples were fixed with 2% osmium tetroxide for 2 hours, dehydrated and embedded in ethoxyline resin at 60 °C for more than 48 hours. Next, thick sections (1000 nm) for further location and thin sections (100 nm) for staining were cut using a Leica EM UC7 ultramicrotome (Leica). Finally, thin sections were mounted into grids and stained with uranyless and lead citrate for observation under TEM (Leica). For quantification, images were collected at 9300 magnification and *g*-ratio was calculated from approximately 300 axons in each group using Image *J* software.

### CAP measurement in the corpus callosum

CAP measurement in the corpus callosum were carried out after 28 days of tMCAO as previously described 50. Briefly, after anesthesia and cardiac perfusion of ice cutting fluid, mice brains were quickly removed and cut into 350 μm coronal slices using a Vibratome (Leica). Brain sections at bregma +0.51 mm and bregma -1.59 mm were put into artificial cerebrospinal fluid (aCSF) pre-gassed with a mixture of 95% O_2_ plus 5% CO_2_, incubated at 34 °C for 0.5 hour and then at RT for 1 hour. The sections were placed in the recording chamber, constantly perfused and submerged with aCSF at 4 to 5 mL/min rate during the whole measurement. The tungsten stimulating electrode (inter-tip distance, 100 μm) was lowered into the corpus callosum approximately 0.5 mm lateral to the midline, the glass recording electrode (3 to 5 MΩ tip resistance when filled with aCSF) was lowered into the corpus callosum approximately 0.75mm from the stimulating electrode. Recordings were acquired using Axon™ pClamp 10 software (Molecular Devices, CA), sampled at 2 to10 kHz and filtered at 1 kHz to differentiate the myelinated (N1) and unmyelinated (N2) components of the CAPs. Data were analyzed using Clampfit 11 software (Molecular Devices, CA). The liquid formulas of aCSF is in the supplemental materials.

### Primary OPC isolation and identification

New born Sprague-Dawley rats within 48 hours were used to isolate primary OPC as described 51. Briefly, the cerebral cortex was gently dissected from the rat pup brain and washed with PBS three times. Then the cerebral cortex was chemically digested and mechanically pipetted to single-cell suspension. After filtering through a 70 μm filter, approximately 1X10^7^ cells were plated on a T75 poly-D-lysine (PDL, Sigma-Aldrich) coated flask and grown for 7-10 days in DMEM with 10% FBS and 1% penicillin-streptomycin antibiotic. When the mixed glial cultures were observed to be confluent, the flasks were first shaken at 180 rpm at 37 °C for 45 to 60 minutes to remove microglia, then the medium was changed and cells were shaken for another 16-20 hours to collect OPCs. The obtained OPCs were further purified by 30-minute culture dish panning, then seeded on PDL coated plates in OPC proliferation medium: Neurobasal-A(Gibco, CA) with 2% B27 (Gibco), 10 ng/mL platelet-derived growth factor AA (PDGF-AA, Gibco), 10 ng/mL basic fibroblast growth factor (bFGF, Peprotech, NJ) and 2 mM glutamine (Gibco).

### OPC proliferation assay

OPCs were seeded to PDL-coated 96-well plate at a density of 1 × 10^4^ per well and cultured in proliferation medium for 1-2 days, then treated with 7.5, 15, 30 μg/mL microglial EVs or the same volume of PBS for 48 hours. The OPC proliferation was evaluated by the cell counting kit-8 (CCK-8, Dojindo, Kumamoto, Japan/ MA0218, Meilunbio, Dalian, CN) according to the manufacturer's instruction. As for OPCs transfected with miRNA mimics, the cell viability was examined 42 hours right after 6 hours of miRNA mimic transfection.

### Oxygen-glucose deprivation (OGD) model *in vitro*

We used the OGD model as previously described 51. Briefly, cultured OPCs were exposed to glucose-free Neurabasal-A medium (Gibco) with oxygen concentration below 0.3% in a hermetically sealed chamber at 37 °C for 2 hours. Then OPCs were returned back to normal condition and treated with EVs for 46 hours. OPCs cultured in proliferation medium without OGD were used as normal control. As for OPCs transfected with miRNA mimics, OPCs were first transfected with mimics for 6 hours and further cultured in proliferation medium for another 18 hours to stabilize the state of OPCs, OPCs were then exposed to OGD for 2 hours and the cell viability was examined 46 hours after OGD.

### OPC differentiation assay

OPCs were plated to PDL-coated glass coverslips in 24-well plate at 6×10^4^ per well for immunostaining and PDL-coated 6-well plate at 6×10^5^ per well for western blot analysis. After culturing in proliferation medium for 2 days, OPCs were treated with PBS, 30 μg/mL M2-EVs or M0-EVs under differentiation medium (Neurobasal-A with 2% B27 and 2 mM glutamine) for 5 days. Medium was exchanged twice a day. Double immunostaining of myelin basic protein (MBP) and NG2 or platelet-derived growth factor receptor α (PDGFR-α), and Western blot analysis of MBP and PDGFR-α expression were performed to examine OPC differentiation. As for OPCs transfected with miRNA mimics, OPCs were first transfected with mimics for 6 hours and further cultured in proliferation medium for another 18 hours to stabilize the state of OPCs, OPCs were then cultured in differentiation medium for 4 days. MBP and myelin oligodendrocyte glycoprotein (MOG) expression were examined using qRT-PCR.

### RNA extraction and qRT-PCR assay

Total RNA was extracted from the ischemic brain or cultured cells using TRIzol LS reagent (Invitrogen, Carlsbad, CA) and reversely transcribed into cDNA by ZymoScript II First Strand cDNA Synthesis Kit (Abconal, Shanghai, CN) according to the manufacturer's instructions. qRT-PCR was performed on a 7900HT (ABI, Foster City, CA) with the following condition: 95 °C for 30 seconds followed by 40 cycles of 95 °C for 5 seconds and 60 °C for 30 seconds. GAPDH was used as the internal reference. For EVs' miRNA, the total miRNA was extracted using miRNeasy Serum/Plasma Kit (Qiagen, GER) and transcribed into cDNA using miRCURY LNA RT Kit (Qiagen, GER). The real-time PCR was performed using miRCURY SYBR Green PCR Kit (Qiagen, GER) according to the kits' instructions. The information of mRNA and miRNA sequences are in the supplementary materials.

### Immunohistochemistry

PFA fixed brain sections or cells were washed three times with PBS for 5 minutes, incubated with 0.3% TritonX-100 for 10 minutes and blocked with 5% bovine serum albumin for 60 minutes at RT, then incubated with primary antibodies at 4 °C overnight. The primary antibodies are used as follows: rat PDGFR-α (1:100; Santa Cruz, CA), mouse PDGFR-α (1:100; eBioscience, CA), NG2 (1:200; Millipore, CA), MBP (1:200; Abcam, Cambridge, UK), SMI32 (1:200; Millipore), adenomatous polyposis coli (APC, 1:200; Millipore), Ki67 (1:200; Millipore/ 1:200, Servicebio, Wuhan, CN), glial fibrillary acidic protein (GFAP, 1:500, Millipore), NeuN (1:200; Millipore), Iba-1 (1:200; WAKO, Osaka, Japan), CD206 (1:200; R&D system, CA), Arginase-1 (Arg-1, 1:50; Santa Cruz), CD31 (1:200; Abcam). After rinsing thrice with PBS, the samples were incubated with corresponding secondary antibodies for 60 minutes at RT. Nucleus was stained with 4, 6-diamidino-2-phenylindole (DAPI, 1:1000; Life Technologies, Mulgrave, VIC, AUS).

### Western blot analysis

Western blot analysis of proteins was conducted as previously described 43, 52. The primary antibodies used were CD206 (1:1000; R&D system), Arginase-1 (Arg-1, 1:500; Santa Cruz), CD63 (1:800; Santa Cruz), TSG101 (1:800; Abcam), Calnexin (1:800; Servicebio), PDGFR-α (1:1000; Santa Cruz), MBP (1:1000; Abcam), β-actin (1:1000; Proteintech, CA), GAPDH (1:1000; Santa Cruz). The semi-quantification of the chemiluminescence signal was calculated using Image *J* software and all gene band gray value was standardized to the expression of β-actin or GAPDH.

### miRNA sequencing of microglia-derived EVs

The miRNA sequencing of M2-EVs and M0-EVs was performed by OE Biotech (Shanghai, CN). Briefly, the total RNAs was extracted from microglial EVs purified from 120 mL of cell medium per sample. A total of 5 μg RNAs were ligated to adaptors at each end, reverse transcribed to cDNA and amplified. The PCR products ranging from 140-160 bp were purified as small RNA libraries and sequenced using the Illumina HiSeq 2500 platform. Reads with 50 bp (single-end read) were generated. Then reads without 3'adaptor and insert tag, or shorter than 15 nt and longer than 41 nt were filtered. The clean read sequences were aligned and subjected to the BLAST search against Rfam v.10.1 (*http://www.sanger.ac.uk/software/Rfam*) and GenBank databases (*http://www.ncbi.nlm.nih.gov/genbank*). Differentially expressed miRNAs were analyzed and identified with the threshold of *p* value < 0.05.

### miRNA mimic and inhibitor transfection

Primary OPCs were transfected with a mimic of indicated miRNA, miRNA negative control (miR-NC) or miR-23a-5p inhibitor after 2 days of seeding using lipo2000 according to the manufacturer's instruction. After 6 hours of transfection, the corresponding OPC proliferation, survival and differentiation assay were carried out.

### Lentiviral vector transduction

Microglia BV2 cells were transduced with RLenti-CMV-EGFP-2A-Puro-U6-mmu-miR-23a-5p-inhibitor and control no-load shRNA lentiviruses (OBiO Technology, Shanghai, CN) at a MOI of 160 for 72 hours in the cell incubator according to the company's instructions. Puromycin (Meilunbio, Dailian, CN) was used to positive select transduced cells and GFP signal was furthered observed under fluorescence microscope to ensure the transduction efficiency.

### Luciferase reporter assay

293T cells were used in luciferase reporter assay. Briefly, the 3'UTRs of genes containing predicted target site of miR-23a-5p were cloned into the pNGM-UTR dual luciferase reporter (Yuanmin Biotechnology Co., Ltd, Shanghai, CN) containing both the Renilla and firefly luciferase (internal control) reporter genes. Mutant constructs were generated with site-directed mutagenesis. 293T cells were seeded into 96-well plates and co-transfected with 0.1 μg luciferase reporters and miR-23a-5p mimic or miR-NC (100 nM). Luciferase activity was examined after 48 hours using the Luc-Pair™ Duo-Luciferase HS Assay Kit (Yuanmin Biotechnology Co., Ltd). According to the Kit's instruction, the relative luciferase activity (Renilla luciferase/firefly luciferase) was calculated to evaluate the regulation of miR-23a-5p on its putative genes.

### Statistical analysis

The sample size was selected based on our previous papers 12, 18, 53 and statistical estimation using power analysis with a power of 0.8 on 2-side and a type I error rate of 0.05. Data were all presented as mean ± standard deviation (SD). Comparisons of means between two groups were analyzed by unpaired Student's t test while among multiple groups by one-way ANOVA followed with Tukey post hoc test using GraphPad Prism 6 (GraphPad Software, USA). Two-tailed *p* value < 0.05 was considered statistically significant.

## Results

### Identification of M2 microglia and microglia-derived EVs

**Figure 1:**
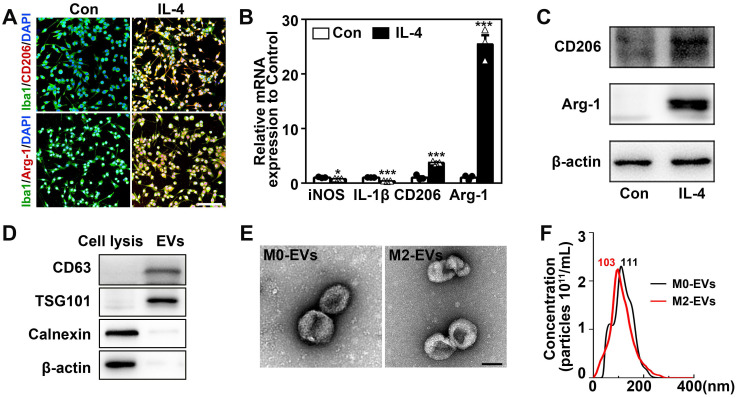
** Identification of M2 microglia and microglia-derived EVs. (A)** Representative immunofluorescent images of IL-4 treated and the control microglia co-stained with Iba1 (green) and CD206/ Arg-1 (red). Scale bar = 200 µm. **(B)** RT-PCR analysis of iNOS, IL-1β, CD206 and Arg-1 expression in the IL-4 treated and the control microglia (n = 3). *, *p* < 0.05; ***, *p* < 0.005. **(C)** Western blots showing CD206 and Arg-1 expression in the IL-4 treated and the control microglia. **(D)** Western blot analysis of CD63, TSG101, Calnexin and β-actin in microglial cell lysis and microglia-derived EVs. **(E)** Representative TEM images of M0-EVs and M2-EVs. Scale bar = 100 nm. **(F)** Nanoparticle tracking analysis of M0-EVs and M2-EVs.

The microglial BV2 cells were used to substitute primary microglia to produce enough EVs for *in vivo* experiments in this study. BV2 cells were polarized to M2 phenotype by treating with 20 ng/mL IL-4 for 48 hours 30, 43. IL-4 treated BV2 cells significantly upregulated M2 microglial marker CD206 and Arg-1 (**Figs. 1A-1C**) and downregulated M1 microglial marker iNOS and IL-1β (**Fig. 1B**, *p <* 0.05). Flow cytometry showed that 89.1% of IL-4 treated microglia were Arg-1^+^ cells and less than 3.6% were CD86^+^ cells (**Fig. S1**). These indicated that we successfully stimulated microglia into M2 phenotype. Then EVs were extracted from IL-4-treated and untreated microglia culture medium (M2-EVs and M0-EVs). The M0-EVs was used as an experimental control. The isolated EVs expressed EVs' marker CD63 and TSG101, whereas the negative control cell lysis did not. Additionally, the endoplasmic reticulum marker, calnexin, and β-actin were not detected in the EVs (**Fig. 1D**). There were no differences between M2-EVs and M0-EVs in the morphology and size distribution of EVs verified by TEM and NTA results (**Figs. 1E-1F**). The purity of our EVs was around1.92x10^9^ particles/1 μg EVs.

### M2-EVs treatment reduced brain atrophy volume, promoted sensorimotor and cognitive functional recovery after 28 days of tMCAO in mice

**Figure 2:**
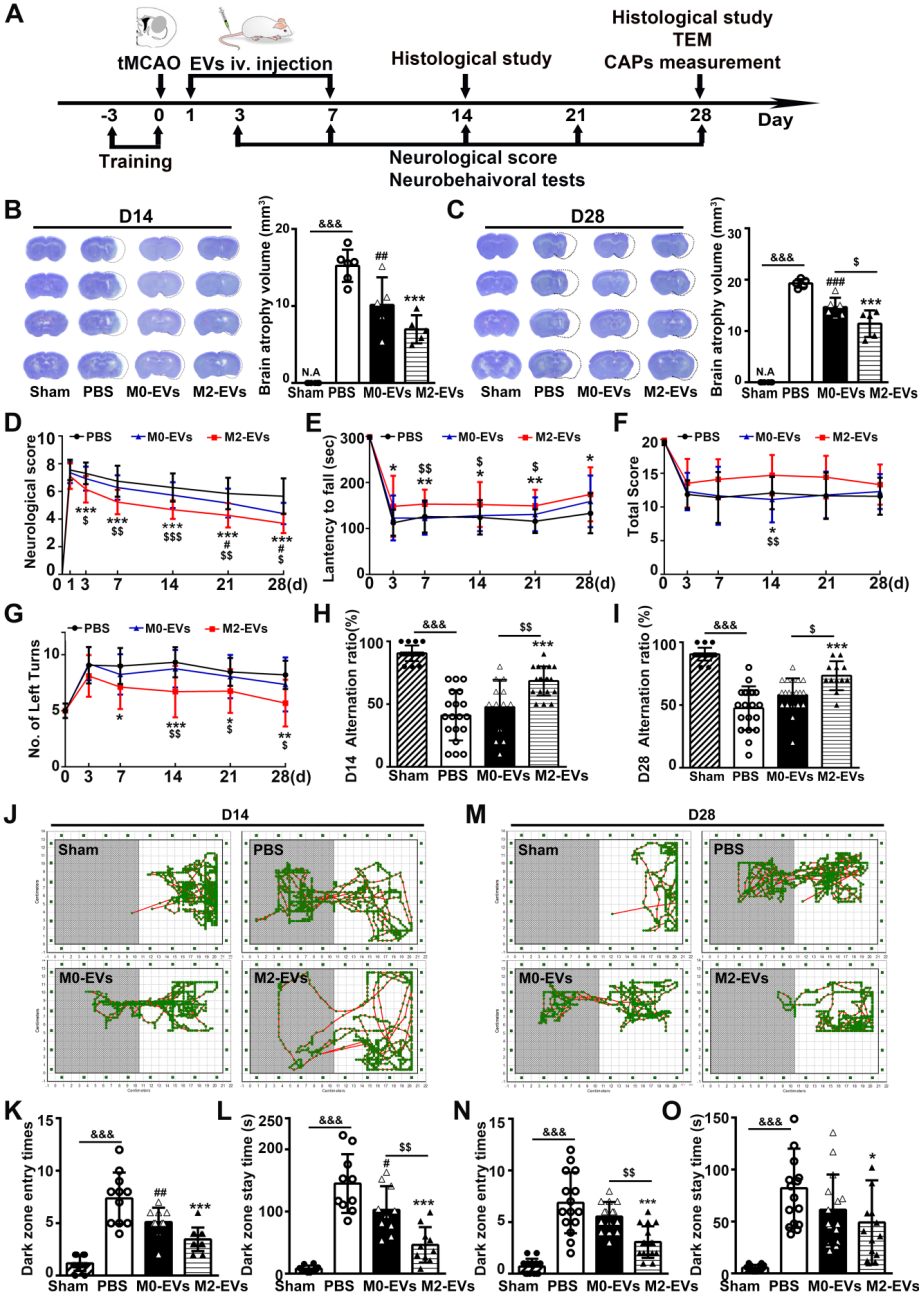
** M2-EVs treatment reduced brain atrophy, promoted sensorimotor and cognitive functional recovery in mice after 28 days of tMCAO. (A)** Diagram of experimental scheme. Mice were sacrificed after 14 days or 28 days of tMCAO. **(B-C)** Representative cresyl violet staining of brain sections and quantification of atrophy volume in the M2-EVs treated, the M0-EVs treated, the PBS control and sham group after 14 days** (B)** or 28 days** (C)** of tMCAO (n = 5). Dashed lines showed the original size of the ischemia hemisphere. **(D-G)** The mNSS **(D)**, rotarod test **(E)**, hanging wire test **(F)** and corner test **(G)** in each group within 28 days of tMCAO (n = 10-16). **(H-I)** The correct alternation ratio of T-maze spontaneous alternation test in each group after 14 days** (H)** and 28 days **(I)** of tMCAO (n = 10-16). **(J-O)** Representative images, bar graphs of dark zone entry times and dark zone stay time of step-through passive avoidance test in each group after 14 days** (J-L)** and 28 days **(M-O)** of tMCAO (n = 10-16). Data are mean ± SD, */^#^/^$^, *p* < 0.05. **/^##^/^$$^, *p* < 0.01, ***/^###^/^&&&^, *p* < 0.005; *, M2-EVs group vs. PBS group; #, M0-EVs group vs. PBS group; $, M2-EVs group vs. M0-EVs group; &, PBS group vs. Sham.

The therapeutic effect of M2-EVs was examined in tMCAO mice. The animal experimental design was depicted in **Fig. 2A**. In the study, a total of 122 ICR mice were used and 32 mice died after tMCAO from day 1 to day 7. The mortality of our tMCAO model was 26%. Twenty-four hours after tMCAO, mice received 200 μL PBS or EVs (100 μg per mouse) once daily for 7 consecutive days via tail vein. As reveal by living animal imaging and immunofluorescent staining, EVs could be detected in the brain from 1 hour up to 48 hours after systemic injection in tMCAO mice, suggesting that the systemically injected microglial EVs could cross the BBB and reach the ischemic region (**Fig. S2**). Additionally, there were no detectable morphological changes and toxic effect in the heart, liver, spleen, lung and kidney 21 days after EVs treatment (**Fig. S3**). The brain atrophy volume was reduced in M2-EVs and M0-EVs groups after 14 and 28 days of tMCAO compared to the PBS group (*p* < 0.05, **Figs. 2B-2C**). Moreover, M2-EVs treatment further reduced brain atrophy volume compared to M0-EVs treatment after 28 days of tMCAO (*p* < 0.05, **Fig. 2C**).

To better determine the effect of M2-EVs on neurological functional recovery after ischemic stroke, we performed neurobehavioral and cognitive function tests at continuous time points within 28 days following tMCAO. mNSS scores decreased in the M2-EVs group after 3, 7, 14, 21 and 28 days of tMCAO compared to the PBS and M0-EVs group (*p* < 0.05, **Fig. 2D**). Moreover, M2-EVs treated mice exhibited better performance in rotarod, hanging wire and corner tests compared to PBS and M0-EVs treated mice (*p* < 0.05, **Figs. 2E-2G**). In the T-maze spontaneous alternation test assessing working memory, M2-EVs group were able to choose better between the novel and familiar arms of the maze after 14 and 28 days of tMCAO as revealed by higher correct alternation ratio compared to the M0-EVs and PBS group (*p* < 0.05, **Figs. 2H-2I**). In the step-through passive avoidance test, the M2-EVs group showed less memory deficits as indicated by fewer dark zone entry times and stay time after 14 and 28 days of tMCAO (*p* < 0.05, **Figs. 2J-2O**). These results suggested that M2-EVs treatment promoted sensorimotor and cognitive functional recovery after tMCAO, while M0-EVs treatment showed negligible effect.

### M2-EVs treatment promoted white matter structural remodeling and functional repair after 28 days of tMCAO in mice

**Figure 3:**
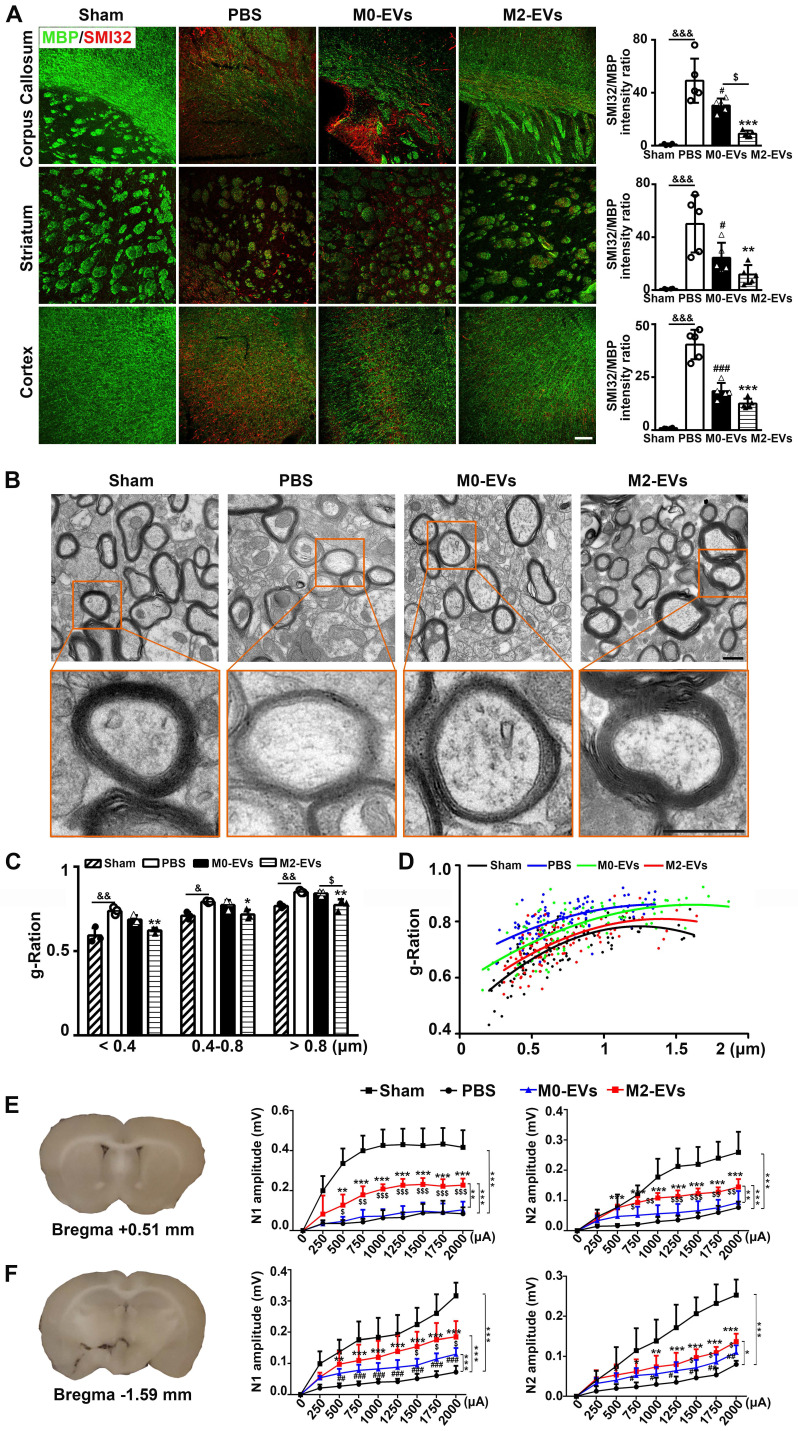
** M2-EVs treatment promoted white matter structural remodeling and function repair in mice after 28 days of tMCAO. (A)** Representative images and quantitative data of demyelinated axon (SMI32^+^, red) relative to myelin (MBP^+^, green) in the corpus callosum, striatum and cortex of the M2-EVs treated, the M0-EVs treated, the PBS treated and sham group after 28 days of tMCAO (n = 4). Scale bar = 100 µm. **(B)** Representative low and high magnification TEM images of myelinated axons in the striatum of each group after 28 days of tMCAO. Scale bar = 0.5 µm. **(C)** The *g*-ratio of myelinated axons with different axon diameter in each group (n = 3, 200-400 axons per mouse). **(D)** Scatter plots of *g*-ratio with respect to different axon diameter in each group. **(E-F)** Representative 350-μm-thick coronal brain sections at bregma +0.51** (E)** mm and bregma -1.59 mm** (F)**, and the N1 (left panel) and N2 (right panel) amplitude of the corpus callosum CAPs in each brain section after 28 days of tMCAO (n = 5-6). Data are mean ± SD, */^#^/^$^/^&^, *p <* 0.05. **/^$$^/^&&^, *p <* 0.01, ***/^###^, *p <* 0.005; *, M2-EVs group vs. PBS group; #, M0-EVs group vs. PBS group; $, M2-EVs group vs. M0-EVs group; &, PBS group vs. Sham.

To investigate whether the promotion of neurobehavioral and cognitive functional recovery was related to white matter structural remodeling and functional repair after M2-EVs treatment, we first examined the extent of white matter remyelination and structural integrity via dual staining of MBP for myelin and SMI32 for demyelinated axons, and TEM analysis of myelin microstructure. The control PBS group exhibited a remarkable loss of MBP^+^ fibers and increase of SMI32^+^ demyelinated axons (resulting in a significant increase of SMI32/MBP immunofluorescent intensity ratio) in the corpus callosum, striatum and cortex after 28 days of tMCAO compared to the sham group (*p* < 0.01, **Fig. 3A**), suggesting ischemic stroke could induce profound white matter injury. Notably, M2-EVs treatment significantly reduced the loss of MBP^+^ fibers and the increase of SMI32^+^ demyelinated axons compared to the PBS group after 28 days of tMCAO (*p* < 0.05, **Fig. 3A**). Although M0-EVs treatment also showed similar effect, the effect was weaker than M2-EVs treatment, especially in corpus callosum (*p* < 0.05, **Fig. 3A**).

We further used TEM to verify the immunostaining results. The *g*-ratio of myelin sheaths is an indicator of myelin thickness. The smaller the *g*-ratio, the thicker the myelin 30. Consistent with MBP and SMI32 staining results, ischemic stroke caused damage to myelin microstructure as confirmed by reduced myelin density (**Fig. 3B**), increased *g*-ratio of small (diameter < 0.4 μm), medium (diameter = 0.4-0.8 μm) and large (diameter > 0.8 μm) axonal fibers (*p* < 0.05, **Fig. 3C**), and the higher myelin sheath *g*-ratio fitting line (**Fig. 3D**) in the PBS group after 28 days of tMCAO. M2-EVs treatment increased myelin density (**Fig. 3B**) and decreased *g*-ratio compared to the PBS group (*p* < 0.05, **Figs. 3B-3C**). While the M0-EVs treatment showed no significant difference with the PBS treatment, and the *g*-ratio of large axonal fibers increased compared to the M2-EVs treatment (*p* < 0.05, **Fig. 3C**). Moreover, the myelin sheath *g*-ratio fitting line of the M2-EVs group was lower than that in the M0-EVs group (**Fig. 3D**). These results collectively suggested that M2-EVs treatment increased myelin sheath thickness, enhanced white matter structural remodeling after tMCAO.

To determine whether the white matter structural remodeling was associated with white matte functional repair, we measured the evoked CAPs in the corpus callosum after 28 days of tMCAO, an electrophysiological examination known as a key method to assess the functional integrity of axons and white matter 50, 54. The early peak (N1 component) of CAPs mainly represents the conduction of myelinated axons, while the later phase peak (N2 component) of CAPs mainly signifies the unmyelinated axons, but the amplitudes of both peaks are the combined results of axonal injury and demyelination 55, 56. As shown in **Figs. 3E-3F**, both N1 and N2 amplitudes of the PBS group decreased at two brain slices compared to sham group (*p* < 0.005), which was consistent with the histological results of myelin loss and axonal injury. M2-EVs treatment increased N1 and N2 amplitudes at both sections compared to the PBS and M0-EVs treatment (*p* < 0.05,** Figs 3E-3F**), indicating that M2-EVs treatment promoted white matter functional repair after tMCAO.

### M2-EVs treatment promoted oligodendrogenesis after 28 days of tMCAO in mice

**Figure 4:**
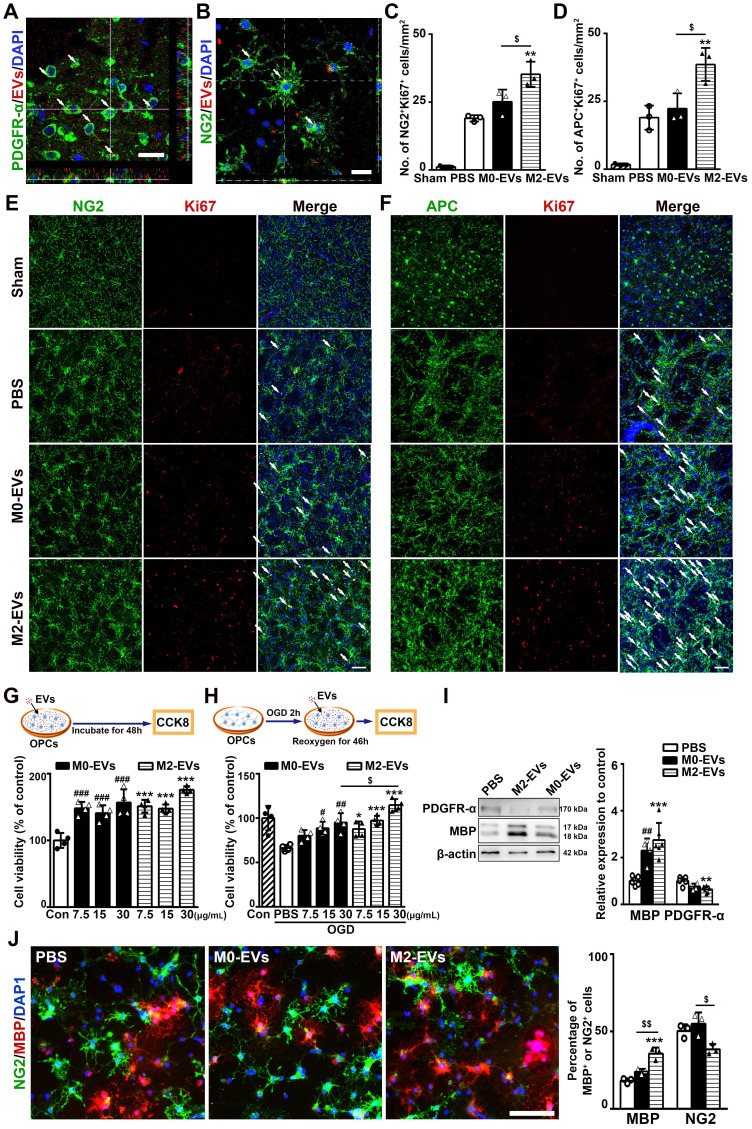
** M2-EVs treatment promoted oligodendrogenesis *in vivo* and OPC proliferation, survival and differentiation *in vitro*. (A)** Representative image showed that the PKH-26 labeled EVs (red) were taken up by PDGFR-α+ OPCs (green) *in vivo*. Arrows indicate EVs located inside the OPCs. Scale bar = 10 µm. **(B)** Representative image showed that PKH-26 labeled EVs (red) were taken up by NG2+ OPCs (green) *in vitro*. Arrows indicate EVs located inside the OPCs. Scale bar = 25 µm. **(C-F)** Quantification and representative immunofluorescent images of NG2/Ki67 (C, E) and APC/Ki67 (D, F) staining in the striatum after 28 days of tMCAO in each group (n = 3). Scale bar = 50 µm. Arrows indicate the NG2+Ki67+ or APC+Ki67+ cells. **(G-H)** Brief experimental diagram and CCK-8 analysis of OPC proliferation (G) and survival (H) that treated with 7.5, 15, 30 µg/mL of M2-EVs or M0-EVs (n = 4). **(I)** Western blot analysis and quantification of PDGFR-α and MBP expression in OPCs after differentiation for 5 days (n = 4-5). **(J)** Representative immunofluorescent images and quantification of NG2 (green) and MBP (red) in OPCs treated with M2-EVs or M0-EVs in the differential medium for 5 days (n = 4). Scale bar = 100 µm. Data are mean ± SD, */#/$, p < 0.05. **/##/$$, p < 0.01, ***/###, p < 0.005; *, M2-EVs group vs. PBS group; #, M0-EVs group vs. PBS group; $, M2-EVs group vs. M0-EVs group.

Since M2 microglia are well-noted in favor of remyelination through regulating OPC function 21, 22, we thus investigated whether M2-EVs promoted white matter repair via regulating the function of OPCs. Firstly, PKH-26 labeled microglial EVs were detected inside the OPCs both *in vitro* and *in vivo*. Sixty minutes after intravenous delivery of PKH-26 labeled microglial EVs in tMCAO mice or 6 hours after incubation with primary cultured OPCs, microglial EVs were localized in the cytoplasm of OPCs (**Figs. 4A-4B, Fig. S4**). These indicated that microglial EVs could enter into OPCs and have the possibility to regulate the function of OPCs.

Then, to investigate the effect of M2-EVs on OPC proliferation and differentiation after tMCAO, we dual stained NG2 and APC with Ki67 to examine the proliferated OPCs and newly formed oligodendrocytes, respectively. Compared to PBS and M0-EVs group, M2-EVs increased the number of NG2^+^Ki67^+^ and APC^+^Ki67^+^ cells after 28 days of tMCAO (*p* < 0.05, **Figs. 4C-4F**), indicating M2-EVs could enhance oligodendrogenesis after ischemic stroke.

### M2-EVs treatment promoted OPC proliferation, survival and differentiation *in vitro*

The isolated primary OPCs expressed two OPC markers PDGFR-α (91.7%) and NG2 (81.5%), but not mature oligodendrocyte marker MBP (**Fig. S5A**).

To examine the effect of M2-EVs on OPC proliferation, OPCs were treated with 7.5, 15 and 30 μg/mL M2-EVs or M0-EVs for 48 hours. CCK-8 assay showed that both M2-EVs and M0-EVs treatment increased the viability of OPCs and displayed a dose dependent manner (*p* < 0.005,** Fig. 4G**). We then evaluated the effect of M2-EVs on OPC survival using OGD model to mimic ischemia-reperfusion injury. As shown in **Fig. 4H**, M2-EVs treatment protected OPCs against OGD injury in a dose dependent pattern compared to M0-EVs treatment, especially at the concentration of 30 μg/mL (*p* < 0.05), suggesting that the protective effect of M2-EVs on OPC survival under OGD was better than M0-EVs. Based on these results, we chose to use 30 μg/mL of EVs in the following *in vitro* studies. We also found that M2-EVs pre-treatment in OGD assay also significantly promoted OPC survival (*p* < 0.05, **Fig. S5B**).

To investigate the effect of M2-EVs on OPC differentiation, we cultured OPCs in differentiation medium with or without the administration of microglial EVs for 5 days. M2-EVs treatment increased MBP expression and decreased PDGFR-α expression compared to the PBS group, but M0-EVs treatment also enhanced MBP expression (*p* < 0.01, **Fig. 4I**). Immunofluorescent results also showed that M2-EVs treatment increased the percentage of MBP^+^ cells compared to the PBS and M0-EVs treatment (*p* < 0.01, **Fig. 4J**), and decreased the percentage of NG2^+^ cells compared to the M0-EVs treatment (*p* < 0.05, **Fig. 4J**).

### MiR-23a-5p was enriched in M2-EVs and enhanced oligodendrogenesis *in vitro*

**Figure 5:**
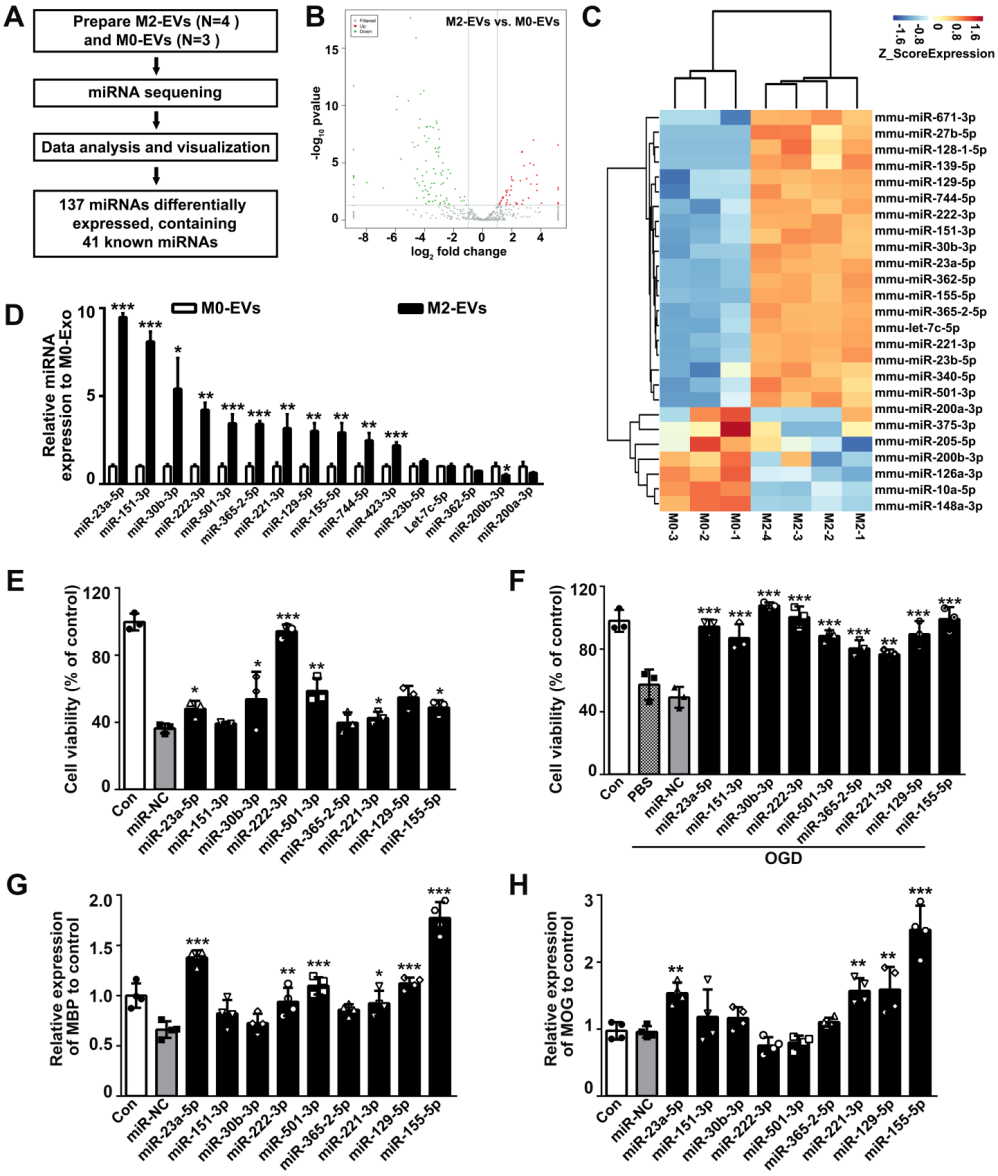
** MiR-23a-5p was enriched in the M2-EVs and enhanced oligodendrogenesis *in vitro.* (A)** Overview of M2-EVs and M0-EVs miRNA sequencing. **(B)** Volcano plot of the miRNA sequencing data. **(C)** Heat map of differentially expressed miRNAs in the M2-EVs and M0-EVs. **(D)** RT-PCR analysis of 16 differentially expressed miRNAs in the M2-EVs and M0-EVs (n = 3). **(E-F)** CCK8 analysis of cell viability in OPCs transfected with a mimic of indicated miRNA or miRNA negative control (miR-NC) under normal condition** (E)** and after OGD** (F)** (n = 3)**. (G-H)** RT-PCR analysis of MBP **(G)** and MOG **(H)** expression in OPCs transfected with a mimic of indicated miRNA or miR-NC after differentiation for 4 days *in vitro* (n = 4). Data are mean ± SD, *, miRNA mimic vs miR-NC in **E-H**; *, *p* < 0.05. **, *p* < 0.01, ***, *p* < 0.005.

As miRNAs are abundant in EVs and mediate cell-to-cell communication, we thus detected the miRNA expression profiles between M2-EVs and M0-EVs using miRNA sequencing (**Fig. 5A**). In all, 137 differentially expressed miRNAs and 41 known miRNAs were identified, among which 45 miRNAs (including 33 known miRNAs) were upregulated, 92 miRNAs (including 7 known miRNAs) were downregulated (**Fig. 5B**). Further clustering of the differentially expressed known miRNAs was shown in a heat map (**Fig. 5C**). The top 14 upregulated and 2 downregulated known miRNAs were verified by qRT-PCR (**Fig. 5D**), and the effects of the top 9 verified upregulated miRNAs on OPC proliferation, survival and differentiation were examined to select our target miRNAs (**Figs. 5E-5H**). Among the verified miRNAs in M2-EVs, the expression level of miR-23a-5p, miR-151-3p and miR-30b-3p were more than 5 times upregulated in M2-EVs than that in M0-EVs (*p* < 0.05, **Fig. 5D**), and only miR-23a-5p increased OPC proliferation (*p* < 0.05, **Fig. 5E**), survival (*p* < 0.05, **Fig. 5F**) and enhanced the expression of MBP (*p* < 0.001, **Fig. 5G**) and MOG (*p* < 0.01, **Fig. 5H**). Although miR-221-3p, miR-129-5p and miR-155-5p were also showed to promote oligodendrogenesis, their expression levels were less than miR-23a-5p in M2-EVs. Thus, we finally focused on miR-23a-5p.

### MiR-23a-5p in M2-EVs was essential in promoting oligodendrogenesis *in vitro* and* in vivo*

**Figure 6:**
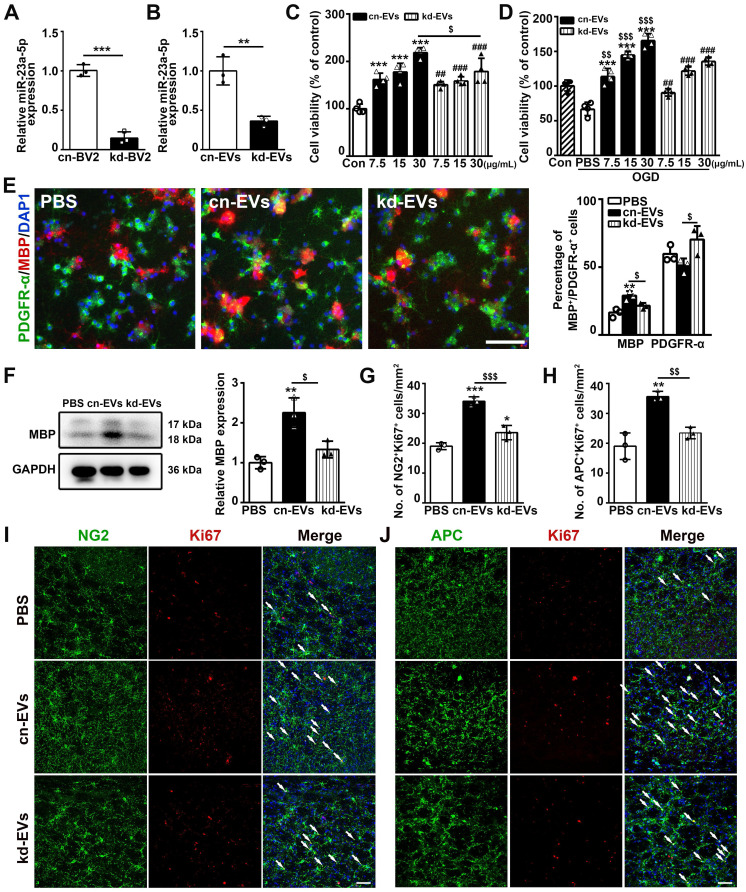
** MiR-23a-5p was essential in promoting oligodendrogenesis afforded by M2-EVs *in vitro* and *in vivo*. (A-B)** RT-PCR analysis of miR-23a-5p expression in cn-BV2 and kd-BV2 (A), cn-EVs and kd-EVs (B) (n = 3). **(C-D)** CCK-8 analysis of OPC viability that treated with 7.5, 15, 30 µg/mL of cn-EVs and kd-EVs under normal condition (C) and after OGD (D) (n = 4). **(E)** Representative images and quantification of PDGFR-α(green) and MBP (red) cells in OPCs treated with cn-EVs and kd-EVs after differentiation for 5 days (n = 3). Scale bar = 100 µm. **(F)** Western blot analysis and quantification of MBP expression in OPCs after differentiation for 5 days (n = 3). **(G-J)** Quantification and representative immunofluorescent images of NG2/Ki67 (G, I) and APC/Ki67 (H, J) staining in the striatum after 28 days of tMCAO in the PBS, cn-EVs and kd-EVs group (n = 3). Scale bar = 50 µm. Arrows indicate the NG2+Ki67+ or APC+Ki67+ cells. Data are mean ± SD, */#/$, p < 0.05; **/$$, p < 0.01, ***/###, p < 0.005; *, cn-EVs group vs. PBS group; #, kd-EVs group vs. PBS group; $, cn-EVs group vs. kd-EVs group.

To further explore whether miR-23a-5p mediated the effect of M2-EVs induced oligodendrogenesis *in vitro* and *in vivo*, we knocked down miR-23a-5p in microglial BV2 cells using lentiviral vector carrying miR-23a-5p-shRNA, the non-load shRNA lentiviral vector was used as a control (kd-BV2 and cn-BV2, **Fig. S6**). EVs were isolated from these microglia treated with IL-4 and named as kd-EVs and cn-EVs, respectively. The qRT-PCR results showed that miR-23a-5p expression in kd-BV2 was decreased to 14.7% compared to cn-BV2 (*p* < 0.005, **Fig. 6A**), and decreased to 36.2% in kd-EVs compared to cn-EVs (*p* < 0.01, **Fig. 6B**), suggesting that miR-23a-5p expression was successfully knocked down in the kd-EVs. To determine whether the lentivirus transduction affected the microglial phenotype, we examined the expression of CD206, Arg-1, iNOS, IL-1β and TNF-α in IL-4 treated BV2, cn-BV2 and kd-BV2 and control cells (**Fig. S7**). Results showed that the IL-4 treated BV2, cn-BV2 and kd-BV2 highly expressed CD206 and Arg-1, lowly expressed iNOS, IL-1β and TNF-α compared to the control (*p* < 0.05, **Fig. S7**), suggesting that the lentivirus transduction did not affect the microglial phenotype.

Then we examined the effects of kd-EVs on oligodendrogenesis *in vitro* and *in vivo*. Kd-EVs treatment partially decreased the cell viability of OPCs under normal condition (*p* < 0.05, **Fig. 6C**) and under OGD (*p* < 0.01, **Fig. 6D**) compared to the cn-EVs treatment. Besides, the kd-EVs pre-treatment in OGD assay also partially reduced the OPC survival (**Fig. S8**). Importantly, kd-EVs treatment reversed the enhancement of OPC differentiation compared to the cn-EVs treatment as indicated by the decreased percentage of MBP^+^ cells, the increased percentage of PDGFR-α^+^ cells (*p* < 0.05, **Fig. 6E**) and the decreased MBP expression (*p* < 0.05, **Fig. 6F**),. Taken together, these results suggested that miR-23a-5p in M2-EVs was essential in promoting oligodendrogenesis *in vitro*.

To examine whether knocking down of miR-23a-5p in M2-EVs reversed the effect of oligodendrogenesis* in vivo*, kd-EVs and cn-EVs were systemically administered to tMCAO mice in the same way. As revealed in **Figs. 6G-6J**, compared to the cn-EVs group, the number of NG2^+^Ki67^+^ and APC^+^Ki67^+^ cells in kd-EVs group was decreased (*p* < 0.01) and nearly similar to the level of PBS group, indicating miR-23a-5p knockdown in M2-EVs reversed the effect of promoting oligodendrogenesis after ischemic stroke.

### MiR-23a-5p in M2-EVs was essential in reducing brain atrophy volume and enhancing functional recovery after tMCAO

Next, we examined whether knocking down of miR-23a-5p in M2-EVs reversed the beneficial effect of M2-EVs on ischemic brain injury and functional recovery. Results showed that kd-EVs treatment partially increased the brain atrophy volume compared to the cn-EVs treatment (*p* < 0.05, **Fig. 6A**). Moreover, kd-EVs treatment reversed the neurobehavioral outcomes (**Figs. 6B-6E**) and cognitive functional recovery compared to the cn-EVs treatment (**Figs. 6F-6H**), suggesting that miR-23a-5p in M2-EVs was essential in reducing ischemic injury and promoting neurological functional recovery after stroke.

### MiR-23a-5p in M2-EVs was essential in promoting white matter structural remodeling and function repair after tMCAO

**Figure 8:**
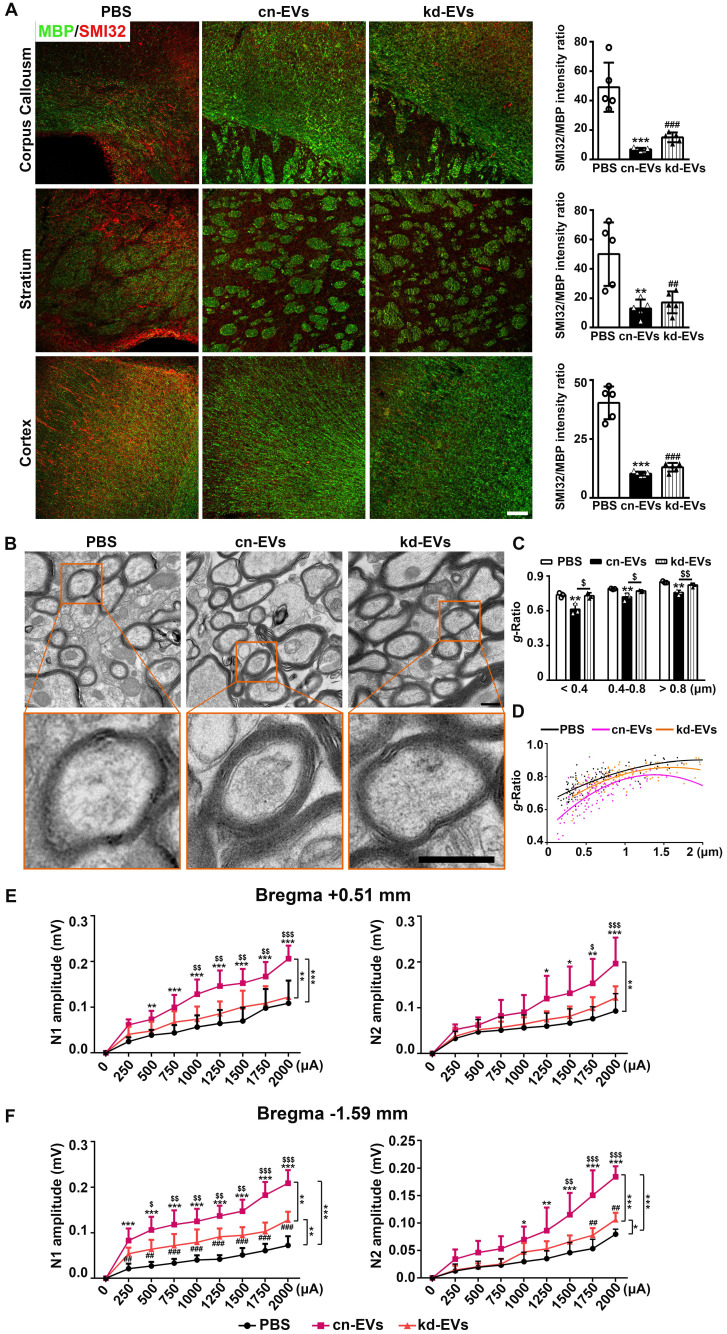
** MiR-23a-5p was essential in promoting white matter structural remodeling and function repair afforded by M2-EVs after tMCAO. (A)** Representative images and quantitative data of demyelinated axon (SMI32^+^) relative to myelin (MBP^+^) in the corpus callosum, striatum and cortex at 28 days after tMCAO (n = 5). Scale bar =100 µm. **(B)** Representative low and high magnification TEM images of myelinated axons in the striatum after 28 days of tMCAO. Scale bar = 0.5 µm. **(C-D)** Quantification of *g*-ratio of myelinated axons **(C)** and scatter plots of *g*-ratio **(D)** with respect to different axon diameter in each group (n = 3, 200-400 axons per mouse). **(E-F)** The N1 (left panel) and N2 (right panel) amplitude of the corpus callosum CAPs in 350-µm-thick coronal brain sections at bregma +0.51 mm **(E)** and bregma -1.59 mm **(F)** in each group, respectively (n = 6). Data are presented as mean ± SD, */^#^/^$^, *p* < 0.05. **/^##^/^$$^, *p* < 0.01, ***/^###^, *p* < 0.005; *, cn-EVs group vs. PBS group; #, kd-EVs group vs. PBS group; $, cn-EVs group vs. kd-EVs group.

We then examined whether knocking down of miR-23a-5p in M2-EVs reversed white matter repair. As shown in **Fig. 8A**, although the difference showed no statistical significance, the ratio of SMI32/MBP immunofluorescent intensity in the kd-EVs group was higher compared to the cn-EVs group (**Fig. 8A**). The *g-ratio*n of axonal fibers with different diameters increased (*p* < 0.05, **Figs. 8B-8C**) and the *g*-ratio fitting line was much closer to the PBS treatment (**Fig. 8D**) in kd-EVs treatment compared to the cn-EVs treatment, suggesting that miR-23a-5p knockdown reduced the capacity of M2-EVs on promoting white matter structural remodeling. Moreover, kd-EVs treatment totally reversed the enhancement of the N1 and N2 amplitudes at bregma 0.51mm sections compared to the cn-EVs treatment (*p* < 0.01, **Fig. 8E**), and partially reversed the promoted effect on N1 and N2 amplitudes at bregma -1.59 mm sections (*p* < 0.01, **Fig. 8F**). These results indicated that miR-23a-5p in M2-EVs played an important role in reducing ischemic injury and improving neurological functional recovery partially via promoting white matter structural remodeling and functional repair after stroke.

### MiR-23a-5p directly targeted Olig3 to promote oligodendrogenesis

**Figure 9:**
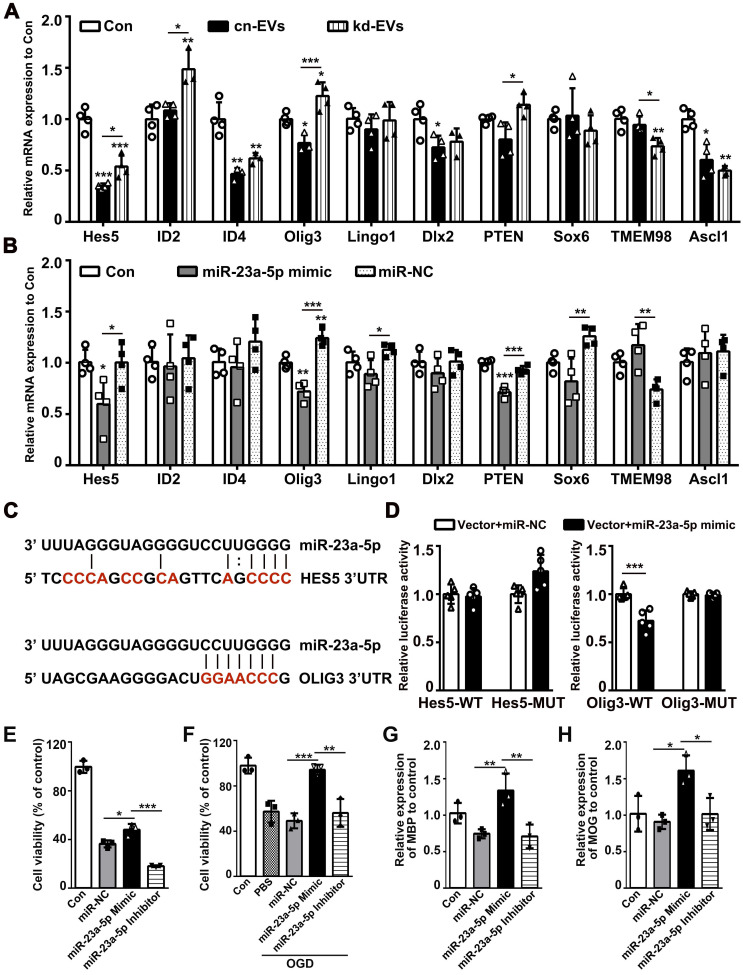
** MiR-23a-5p directly targeted Olig3 to promote oligodendrogenesis. (A-B)** RT-PCR analysis of Hes5, ID2, ID4, Olig3, Lingo1, Dlx2, PTEN, Sox6, TMEM98 and Ascl1 expression in the control, cn-EVs and kd-EVs groups **(A)**, the control, miR-23a-5p mimic and miR-NC groups **(B)** after 4 days of OPC differentiation *in vitro* (n = 3-4). **(C)** Predict binding sites of miR-23a-5p in the 3'UTRs of HES5 and OLIG3 genes. Sites mutated as controls in the luciferase reporter assay are shown in red. **(D)** Dual luciferase reporter assay for the direct and specific interaction of miR-23a-5p with the 3'UTRs of Hes5 and Olig3 (n = 5). **(E-F)** CCK8 analysis of cell viability in OPCs transfected with miR-NC, miR-23a-5p mimic or miR-23a-5p inhibitor under normal condition** (E)** and after OGD** (F)** (n = 3)**. (G-H)** RT-PCR analysis of MBP **(G)** and MOG **(H)** expression in OPCs transfected with miR-NC, miR-23a-5p mimic or miR-23a-5p inhibitor after 4 days of differentiation *in vitro* (n = 3). Data are presented as mean ± SD, *, *p* < 0.05; **, *p* < 0.01; ***, *p* < 0.005.

To find the exact target gene regulated by miR-23a-5p in oligodendrogenesis and white matter repair, we first scanned the predicted miR-23a-5p target genes in the TargetScan, miRDB and miRWalk database, then combined with genes reported to modulate the function of OPCs. The screened genes including Hes5, ID2, ID4, Olig3, Lingo1, DLX2, PTEN, SOX6, TEME98 and Ascl1 were examined in kd-EVs, cn-EVs, miR-23a-5p mimic and miR-NC treated OPCs by qRT-PCR. Results showed that Hes5 and Olig3 were decreased in kd-EVs and miR-23a-5p mimic group (*p* < 0.05, **Figs. 9A-9B**). So, we examined whether Hes5 and Olig3 were directly targeted by miR-23a-5p using dual-luciferase reporter assay. Luciferase reporter assay showed that miR-23a-5p mimic significantly inhibited the luciferase activity in 293T cells transfected with the Olig3 but not Hes5 3'-UTR plasmid compared to miR-NC (*p* < 0.001, **Figs. 9C-9D**), suggesting Olig3 was the direct target gene of miR-23a-5p. To better explore the effect of miR-23a-5p on oligodendrogenesis, we transfected miR-23a-5p mimic and inhibitor into OPCs. MiR-23a-5p mimic increased but miR-23a-5p inhibitor decreased or showed no effect on OPC proliferation, survival and differentiation (*p* < 0.05, **Figs. 9E-9H**). These results indicated that miR-23a-5p possibly directly targeted Olig3 to promote oligodendrogenesis.

## Discussion

In the present study, we demonstrated that M2-EVs treatment reduced ischemic brain injury, promoted sensorimotor and cognitive function outcomes, enhanced oligodendrogenesis, white matter structural remodeling and functional repair after ischemic stroke. Furthermore, M2-EVs promoted OPC proliferation, survival and differentiation *in vitro*. We provided deeper insights into the underlying mechanism that miR-23a-5p enriched in M2-EVs was essential for the therapeutic effect on white matter remodeling and repair after ischemic stroke possibly via directly targeting olig3 to promote oligodendrogenesis (**Fig. 9**).

EVs derived from microglia/macrophage showed multiple therapeutic effects after injury. Actually, our group has been studying the mechanisms of M2-EVs in ischemic stroke 25, 43. We found that in the acute phase (3 days) of tMCAO, M2-EVs protected neuron against apoptosis via transferring EVs' miR-124 to neurons and inhibiting ubiquitin-specific protease 14 expression 43. In the subacute phase (14 days) of tMCAO, M2-EVs could inhibit astrocyte proliferation and glia scar formation through transferring miR-124 to astrocytes and directly targeting signal transducer and activator of transcription 3 25. Additionally, M2-EVs restored protective microglia/macrophages functions via inhibiting their senescence after permanent MCAO 42. In the present study, we investigated the mechanism of M2-EVs on white matter repair in the chronic phase of tMCAO. We demonstrated that M2-EVs promoted oligodendrogenesis through transferring EVs' miR-23a-5p and possibly via directly targeting Olig3.

Our study supported that M2-EVs improved neurological functional recovery and white matter repair following tMCAO by increasing OPC proliferation, survival and differentiation. First, M2-EVs treatment reduced brain atrophy volume, improved sensorimotor and cognitive functional recovery as evidenced by the better performance in mNSS score, rotarod test, hanging wire test, corner test, T-maze spontaneous alternation test and step-through passive avoidance test. Secondly, the reduction of SMI32/MBP intensity ratio, the increase of myelin thickness and density, the increase of N1 and N2 amplitudes at two brain sections were observed in M2-EVs treatment than M0-EVs and PBS treatment. Thirdly, OPCs could uptake microglial EVs* in vivo* and *in vitro*. Finally, M2-EVs treatment protected OPCs against ischemia-reperfusion injury *in vitro*, increased OPC proliferation and enhanced OPC differentiation *in vitro* and *in vivo*, which could contribute to white matter structural remodeling and functional repair observed *in vivo*. These results provide clear evidence that microglia could modulate OPC function by EVs and M2-EVs treatment has a great potential in demyelinating diseases including ischemic stroke.

MiRNAs in EVs are well-documented to meditate the functions of EVs and could show dramatic regulation in recipient cells 57. Intriguingly, miRNAs are critical regulators of OPC functions by modulating various stage-specific genes not only in development but also during remyelination in diseases 31-33. Using miRNA sequencing and qRT-PCR verification, we found miR-23a-5p was enriched in M2-EVs and promoted OPC proliferation, survival and differentiation among the verified upregulated miRNAs. Furthermore, we demonstrated that miR-23a-5p knockdown in M2-EVs reduced OPC proliferation and survival, abolished the effect on OPC differentiation and oligodendrogenesis. Consistently, miR-23a-5p knockdown partially reversed the beneficial effects of M2-EVs on reducing ischemic injury, promoting neurological functional recovery, white matter structural remodeling and functional repair after cerebral ischemia. These suggested that miR-23a-5p in M2-EVs was essential for the therapeutic effect of promoting oligodendrogenesis and white matter repair.

Under normal culture condition, the cell viability in miR-NC was decreased compared to the control, which could be due to the toxic effect of lipo2000. It is noted that OPCs transfected with these miRNA mimics showed better cell viability under OGD than under normal condition. One reason could be that the examining time point of OPC viability was different. Another reason could be that compared to normal condition, OPCs may be more sensitive to these miRNA mimics since OPCs were stimulated by hypoxia and starvation under OGD 58. Nevertheless, the transfection of these miRNA mimics did not significantly reduced the cell viability of OPCs compared to miR-NC. It was noted that miR-23a-5p knockdown in M2-EVs mainly inhibited OPC maturation, which supported the concept that the effects of EVs could not be simply ascribed to only one miRNA inside. Indeed, our study also found that other miRNAs in M2-EVs such as miR-221-3p, miR-129-5p and miR-155-5p could promote OPC proliferation, survival and maturation. This may explain why kd-EVs treatment had no significant difference compared with cn-EVs group in the ratio of SMI32/MBP fluorescence intensity. Besides, recent studies showed that the lipids and transmembrane protein TNF in M2 microglial EVs promoted OPC migration and differentiation 30. Different miRNAs and contents in EVs may prone OPC to different developing states under different culture conditions 30, 42, which may be the reason that M0-EVs treatment also enhanced MBP expression. Also, it was note that the decreased extent of miR-23a-5p level in kd-BV2 was higher than that in kd-EVs, which indirectly suggested that miR-23a-5p was selectively enriched in M2-EVs.

Olig3 is a member of basic helix-loop-helix transcription factors that expresses in neurons and glial cell lineages 59. While the function of Olig1 and Olig2 on oligodendrogenesis in the development and diseases are well documented 60, little is known about the function of Olig3 on oligodendrogenesis, especially in diseases condition. In the development, Olig3 was shown to be essential for the specification of dl1-dl3 interneurons 61 and regulate the production of GABAergic neurons 62, 63. Our study demonstrated that miR-23a-5p directly targeted Olig3, inhibited Olig3 expression during OPC differentiation* in vitro*, promoted oligodendrogenesis *in vitro* and *in vivo*, suggesting that the enhanced oligodendrogenesis after stroke was possibly associated with the inhibition of olig3. The specific function and mechanism of Olig3 on oligodendrogenesis in ischemic stroke need further and deeper investigation.

In our study, microglial EVs were administrated once daily for seven consecutive days from day 1 after tMCAO via tail vein. Since clinical interventions are always behind the stroke onset, we did not choose to inject immediately after tMCAO, this time window may be better for EVs treatment translation in clinic. Given that in ischemic stroke, the increasing loss of oligodendrocytes was observed as early as 24 hours after ischemia, and the number of oligodendrocytes begun to increase within the acute phase (7 days) after stroke, which was attributed to the activation, proliferation and differentiation of endogenous OPCs^10^, ^11^. As we know, the number of survived oligodendrocytes and newly formed mature oligodendrocytes from OPCs is the key factor to preserve myelin integrity and normal axonal function, which contributes to white matter repair 4, 11. In order to investigate the effect and the effect duration of early M2-EVs intervention on the survival, proliferation, differentiation of OPCs and white matter repair, we chose to administrate M2-EVs from day 1-7 after tMCAO and evaluated the therapeutic effects on white matter remodeling 14 and 28 days after ischemic stroke. Previous studies and our published work have proven that the continuous EVs delivery did not provoke any inflammatory, immune responses and visible tissue toxicity 43, 64. Indeed, we did not find any detectable morphological changes and toxic effect in the heart, liver, spleen, lung and kidney 21 days after EVs treatment.

Notably, the distribution and metabolism of EVs in mice are important issues that need more basic investigations, which were studied recently. For example, biodistribution study of EVs showed that the majority of systemically administrated EVs were trapped in the liver, lungs, spleen, kidney, stomach and intestines 65, but EVs were accumulated in the ischemic brain as early as 1 hours post injection and largely cleared from the brain by 24 hours after ischemia 66. The clearance of EVs from blood began 1 hour and lasted 6 hours post intravenous injection 67. To dynamically detect EVs' mRNA translations, EVs' mRNA cargos were fused with gaussia luciferase reporter gene. Results showed that the gaussia luciferase mRNA translation occurred within 1 hour, peaked at 12 hours and gradually decreased from 12 to 72 hours post EVs' delivering to recipient cells 68. In our study, we found that compared to the control group, M2-EVs could be detected in the brain from 1 hour up to 48 hours after injection. Studies showed a carefully timed application of EVs in batches was found more effective than a single total application 69. Therefore, we decided to give EVs once daily.

Taken together, our study demonstrated that M2-EVs promoted neurological functional recovery, white matter structural remodeling and functional repair through enhancing oligodendrogenesis via EVs' miR-23a-5p possibly by directly targeting Olig3 after ischemic stroke, suggesting that M2-EVs could be a novel therapeutic strategy for promoting white matter remodeling and repair in stroke and demyelinating diseases.

## Supplementary Material

Supplementary methods, figures and tables.

## Figures and Tables

**Figure 7:**
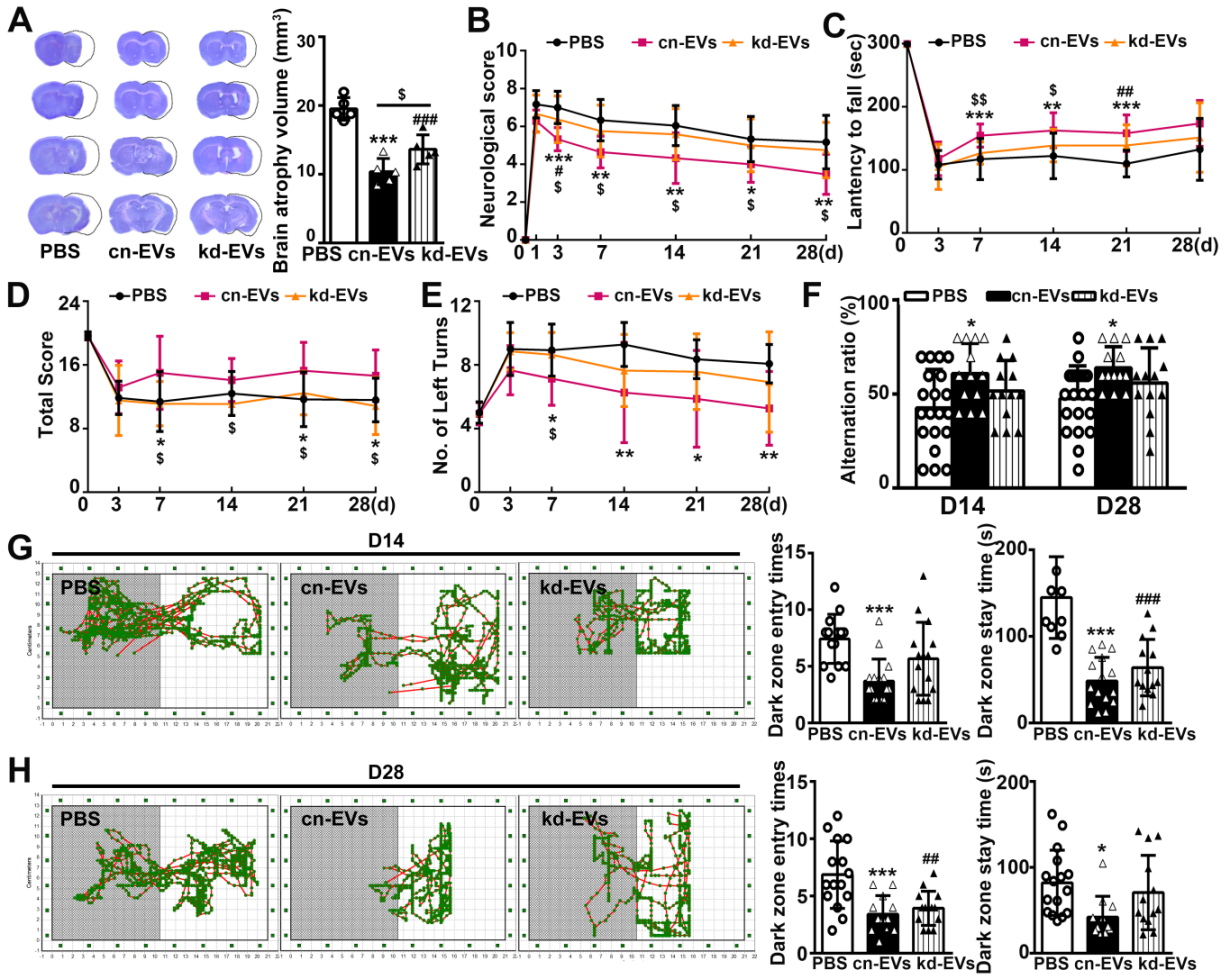
** MiR-23a-5p was essential in reducing brain atrophy volume and enhancing functional recovery afforded by M2-EVs after tMCAO. (A)** Cresyl violet staining of brain sections and quantification of atrophy volume in the cn-EVs, the kd-EVs and the PBS treated groups after 28 days of tMCAO (n = 5). Dashed lines showed the original size of the ischemia side. **(B-E)** The mNSS **(B)**, rotarod test **(C)**, hanging wire test **(D)** and corner test **(E)** in each group at different time points after tMCAO (n = 10-16). **(F)** T-maze spontaneous alternation test at 14 and 28 days after tMCAO (n = 10-16). **(G-H)** Representative images, dark zone entry times and dark zone stay times of step-through passive avoidance test at 14 **(G)** and 28 **(H)** days after tMCAO (n = 10-16). Data are mean ± SD, */^$^/^#^, *p <* 0.05; **/^$$^/^##^, *p <* 0.01, ***/^###^, *p <* 0.005; *, cn-EVs group vs. PBS group; #, kd-EVs group vs. PBS group; $, cn-EVs group vs. kd-EVs group.

## Abbreviations

aCSF: artificial cerebrospinal fluid
APC: adenomatous polyposis coli
Arg-1: arginase-1
BBB: blood-brain barrier
cn-EVs: extracellular vesicles from miR-23a-5p knock down M2 microglia
IL-4: Interleukin 4
kd-EVs: extracellular vesicles from miR-23a-5p knock down M2 microglia
M0-EVs: untreated microglia-derived extracellular vesicles
M2-EVs: M2 microglia-derived extracellular vesicles
MBP: myelin basic protein
mNSS: modified neurological severity score
MOG: myelin oligodendrocyte glycoprotein
NTA: nanoparticle tracking analysis
OL: oligodendrocyte
OPC: oligodendrocyte precursor cell
PTEN10: phosphatase and tension homolog on chromosome 10
TEM: transmission electron microscope
TGF-α: transforming growth factor α
tMCAO: transient middle cerebral artery occlusion
TNF-α: tumor necrosis factor α
TSG 10: tumor of susceptibility gene 101
