# Supplementary material for: M2 microglia-derived extracellular vesicles promote white matter repair and functional recovery via miR-23a-5p after cerebral ischemia in mice
Source: Theranostics. 2022 Apr 24;12(7):3553–73. doi: 10.7150/thno.68895 (PMC9065182; doi:10.7150/thno.68895)
Supplement: Supplementary file 1 — Supplementary methods, figures and tables. [file thnov12p3553s1.pdf]

Supplemental materials

Supplemental methods

Modified neurological severity score (mNSS)

mNSS ranging from 0 to 18 score was assessed from four aspects [1]: the motor function (0-6) including lifting the mouse by the tail (0-3) and walking on the floor (0-3), the sensory function (0-2) including superficial and deep sensation, the balance function (0-6) including walking on a beam, the reflex reaction and muscular tension (0-4).

Rotarod test

The rotarod test was performed as previous described [2, 3]. Briefly, the mice were trained on a rotating rod at a speed of 20 rpm/min for 5 minutes and three times a day for 3 consecutive days before the tMCAO surgery. At each examine point, the mice were first placed on the rotarod for adaption, then the rotarod was accelerated to 40 rpm/min within 90 seconds and the total test time was 5 minutes. The test trail was repeated three times with an interval more than 15 minutes, the time mice stay on the rod (latency to fall) were recorded and the average time was used for data analysis.

Hanging wire test

Hanging wire test was performed as previously described [2, 4]. Briefly, the mice were gently placed on a steel wire (55 cm long and 2 mm thick) for adaption, then placed in the middle of the wire with only two fore limbs and they would manage to climb to one side of the wire. The times mice reached

one side or fell off the wire were recorded. Once the mice arrived or fell, they would be placed back to the middle of the wire immediately until the 3 minutes test time finished. The total score was calculated by adding the times of arrivals and subtracting the times of falls from the initial score of 10.

Corner test

The mice were subjected to 10 trails of corner test, during which the left or right turns were recorded [5]. Briefly, the mouse was placed to a 30° angle corner formed by two boards in a quiet and dark room, it would turn back when entering into the corner. Only when the mouse moved forward, upward and put one of the fore limbs onto the board then turn back was considered as a trail. Normal mice would turn to either side equally, while the ischemic mice would tend to turn to the lesion side, which is the left side in our study.

T-maze

T-maze was performed to exam the working memory function of the mice [6]. At 14 or 28 days after tMCAO, the mouse was placed in the start area and free to choose a goal arm. After the mouse entering the chosen arm, quickly and quietly closed the door and confined the mouse in the chosen arm for 15 seconds, then took it out to a cage, rubbed the maze with 75% alcohol, and replaced it in the start area to allow the mouse choose between the familiar and novel arms, if the mouse chose the novel arms it was considered as a correct spontaneous alternation. The alternations were recorded in 10 trails for each mouse.

Step-through passive avoidance test

Step-through passive avoidance test was performed as previously described [7]. Briefly, one day before the examine time point, the mouse was placed into the light zone of the smart cage (AfaSci Research Laboratories, Redwood City, CA) with free access to the dark zone. When mouse entered into the dark zone and stayed more than 2 seconds, it received an electric foot shock. After 24 hours, the mouse was replaced into the light zone without electric stimulation and recorded the moving trace for 5 minutes. The dark zone entry times and dark zone stay time were analyzed automatically using the CageScore 2.6 software.

The liquid formulas of aCSF

The detailed liquid formulas of aCSF was as follows: 119 mmol/L NaCl, 2.5 mmol/L KCl, 1 mmol/L NaH_2PO_4, 1.3 mmol/L $MgSO_4$, 2.5 mmol/L $CaCl_2$, 26.2 mmol/L $NaHCO_3$, 10 mmol/L glucose; pH 7.4 [8].

Supplemental Figures

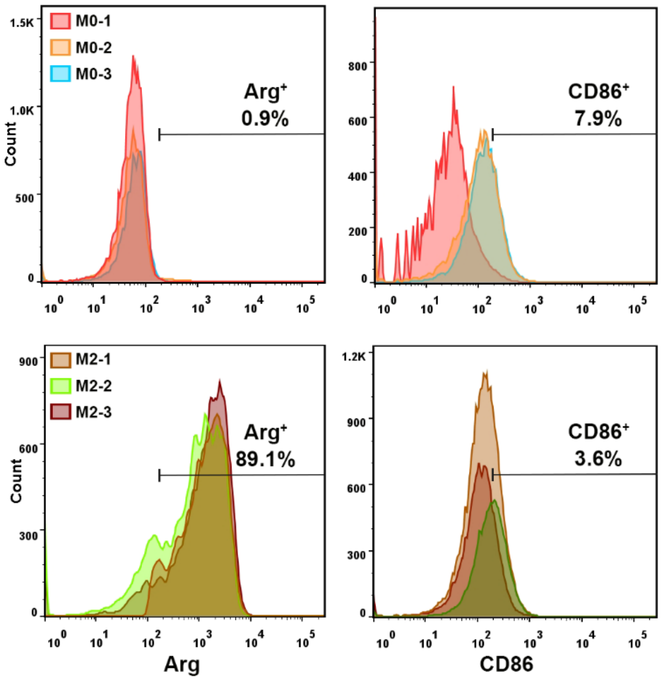

Figure S1. Identification of M2 microglia using flow cytometry.

Flow cytometry showing that that 89.1% of IL-4 treated microglia were Arg-1+ cells and less than 3.6% were CD86+ cells.

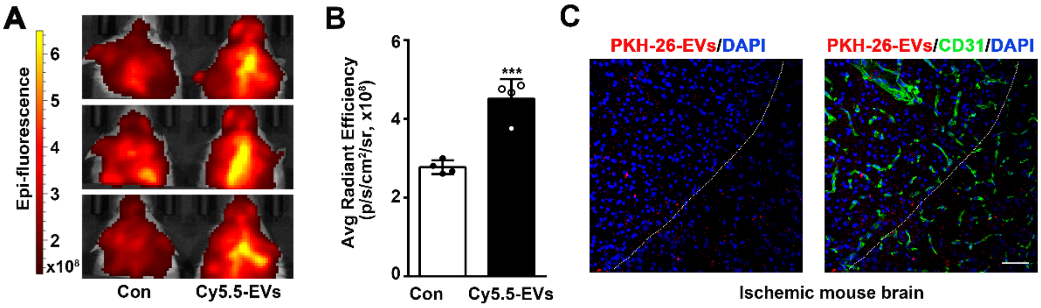

Figure S2. Systemically injected microglia-derived EVs crossed the BBB after tMCAO in mice.

(A) Representative living animal images showed that the cy5.5-labeled EVs (Cys.5-EVs) were accumulated in the ischemic brain after tail vein injection at

24 hours after tMCAO. **(B)** Quantification of the average radiant efficiency in the control and Cy5.5-EVs groups from A (n = 4). **(C)** Representative images of the CD31 (red) staining 1 hour after PKH-26-EVs injection in the ischemic mouse brain. Scale bar = 50 μm. Dash line indicates the border of the ischemic core and peri-infarct region. Data are mean ± SD, ***, $p < 0.005$.

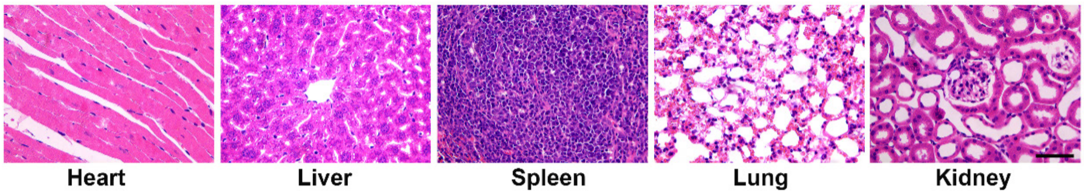

Figure S3. HE staining of different organs 21 days after 7 consecutive days of systemic injection of microglia-derived EVs in tMCAO mice.

Representative HE images showed no detectable morphological changes and toxic effect in the heart, liver, spleen, lung and kidney 21 days after 7 consecutive days of tail injection of microglia-derived EVs in tMCAO mice. Scale bar = 50 μm.

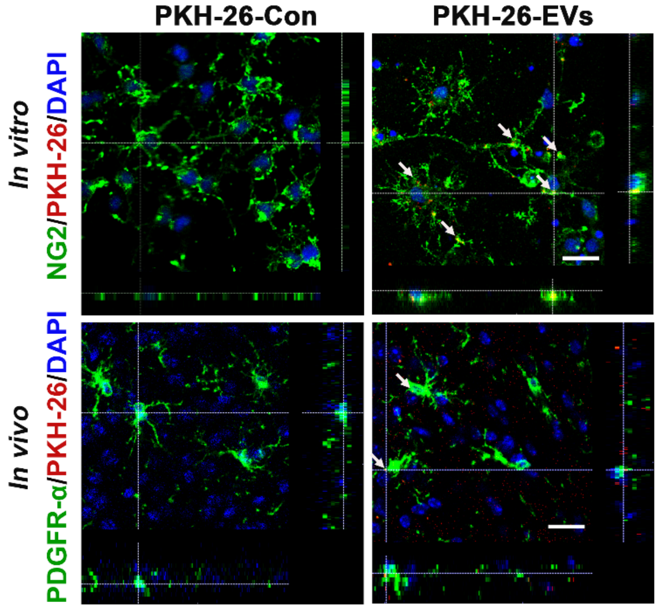

Figure S4. OPCs uptook microglia-derived EVs *in vitro* and *in vivo*.

Representative immunofluorescent images showed that compared to the PKH-26-EVs group, no PKH-labelled red dots were detected in the cytoplasm of OPCs in the PKH-26-Con group both *in vivo* and *in vitro*. Scale bar = 20 μm.

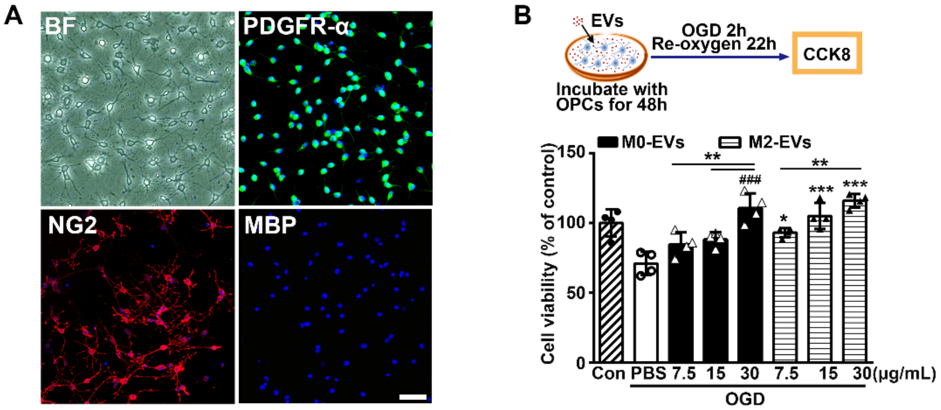

Figure S5. OPC identification and the effect of M2-EVs pre-treatment on OPC survival.

(A) Bright field and immunofluorescent images of primary OPCs cultured for 2 days staining with PDGFR-α (green), NG2 (red) and MBP (green). Nuclear was stained with DAPI (Blue). Scale bar = 50 μm. **(B)** Brief experimental diagram and CCK-8 analysis of OPC survival that pre-treated with 7.5, 15, 30 μg/mL of M2-EVs or M0-EVs for 48 hours (n = 4). Data are mean ± SD, *, p < 0.05. **, p < 0.01, ***/$^{###}$, p < 0.005; *, M2-EVs group vs. PBS group; #, M0-EVs group vs. PBS group.

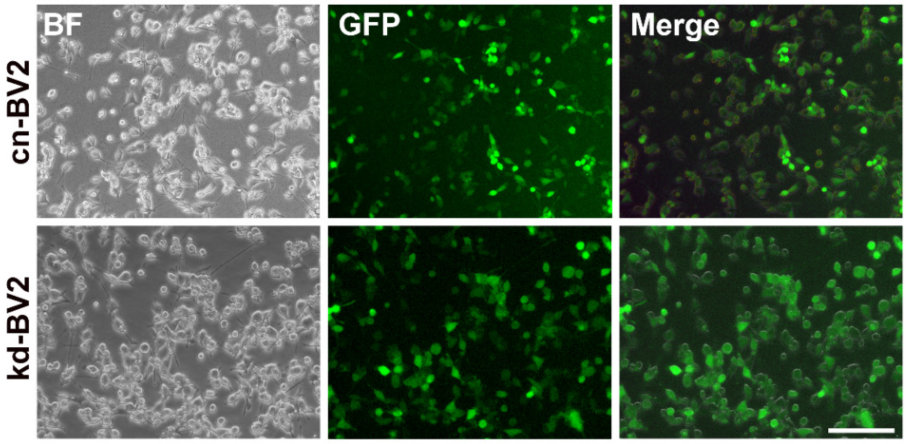

Figure S6. Images of lentivirus transduced BV2 cells.

Representative bright field (BF), immunofluorescent and merge images of microglia BV2 cells transduced with miR-23a-5p-inhibitor (kd-BV2) or control no-load shRNA lentiviruses (cn-BV2). Scale bar =100 μm.

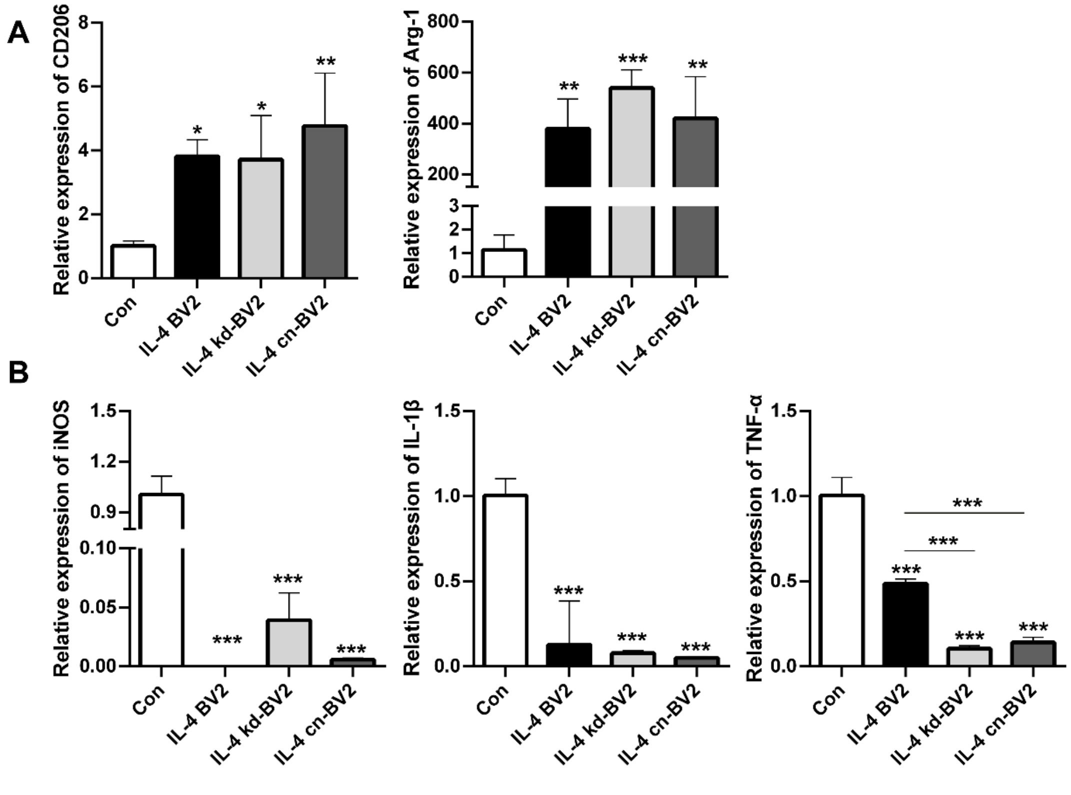

Figure S7. Identification of M2 microglial phenotype in IL-4-treated kd-BV2 and cn-BV2 cells.

RT-PCR analysis of CD206, Arg-1, iNOS, IL-1β and TNF-α expression in the IL-4 treated BV2, kd-BV2, cn-BV2 and the control BV2 cells (n = 3). *, $p < 0.05$; **, $p < 0.01$; ***, $p < 0.005$.

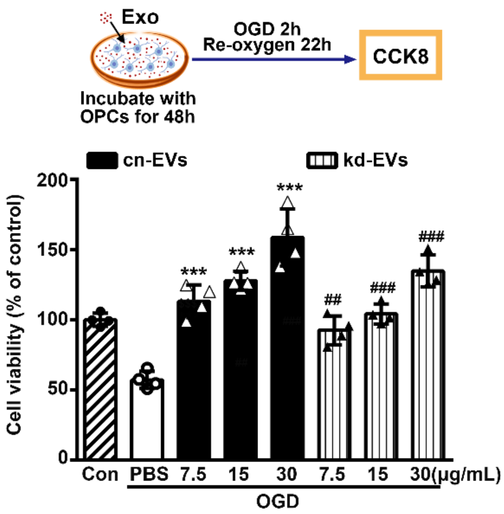

Figure S8. The effect of kd-EVs pre-treatment on OPC survival.

Brief experimental diagram and CCK-8 analysis of OPC survival that pre-treated with 7.5, 15, 30 μg/mL of cn-EVs or kd-EVs for 48 hours (n = 4). Data are mean ± SD, **/##, $p < 0.01$, ***/###, $p < 0.005$; *, cn-EVs group vs. PBS group; #, kd-EVs group vs. PBS group.

Supplemental tables

Table S1. The sequence of mRNAs.

mRNA	Forward primer	Reverse primer
mouse Arg-1	CATATCTGCCAAAGACATCGTG	GACATCAAAGCTCAGGTGAATC
mouse CD206	CAGGTGTGGGCTCAGGTAGT	TGTGGTGAGCTGAAAGGTGA
mouse IL-1β	GAAATGCCACCTTTTGACAGTG	TGGATGCTCTCATCAGGACAG
mouse iNOS	CAGAGGACCCAGAGACAAGC	TGCTGAAACATTTCCTGTGC
mouse TNF-α	TAGCCAGGAGGGAGAACAGA	CCAGTGAGTGAAAGGGACAGA
mouse GAPDH	GATGGTGAAGGTCGG TGT GA	TGA ACTTGCCGTGGG TAG AG.
rat GAPDH	GATGGTGAAGGTCGGTGTGA	TGAACTTGCCGTGGGTAGAG
rat MBP	ACCACTCTGGAAAGCGAGAATTAGC	ACTGTCTTCTGAGGCGGTCTGAG
rat MOG	GAAGAAGCCGCCGTGGAGTTG	GCAGGAGCAGCATAGGCACAAG

rat PDGFR-α	CTGGTCTTATGGCGTTCTGCTCTG	CTGGTGGCATGGTCGGGTTTG
rat Hes5	TGGCGGTGGAGATGCTCAGTC	GCTCAATGCTGCTGTTGATGCG
rat ID2	CCCACTATCGTCAGCCTGCA	GTCCGTGTTCAGGGTGGTCA
rat ID4	CCATCCCGCCCAACAAGAAAGTC	AGCAGAGCAGGGTGCGTCTC
rat Olig3	CAAGCTCTCCAAGATCGCCACTC	CTCGCCAACCAACCTCTTCATCTC
rat Lingo1	CAGGTACAGGACAACGGCAC	GGCTGGTTGGAGATGAAGGC
rat Dlx2	CAACGAGCCCGACAAGGAAGAC	CTGGAGTAGATGGTGCGTGGTTTC
rat PTEN	TTGAAGACCATAACCCACCACAGC	CATTACACCAGTCCGTCCTTTCCC
rat Sox6	GAGTCGGGAGCGGGAAATAATGAAC	CGTATCCACCACATCGGCAAGG

**mRNA**	**Forward primer**	**Reverse primer**
rat TMEM98	GCCAAACGGATTAGCCCAAGAGTG	AGTGACTAACGGACAGGAGCAGAG
rat Ascl1	AGCAGCAACAGCAGCAGCAG	AGCGCATCAGTTCGGGAGAGG

Table S2. The sequence of miRNAs

**Mouse miRNA**	**Primer Sequence**
miR-23a-5p	GGTTCCTGGGGATGGGATTT
miR-151-3p	GCTAGACTGAGGCTCCTTGAGG
miR-30b-3p	CGCTGGGATGTGGATGTTTACGTC
miR-222-3p	AGCTACATCTGGCTACTGGGTCT
miR-501-3p	AATGCACCCGGGCAAGGATTT
miR-365-2-5p	TAGGGACTTTCAGGGGCAGC
miR-221-3p	CCAGCTACATTGTCTGCTGGGTTTC
miR-129-5p	CTTTTTGCGGTCTGGGCTTGC
miR-155-5p	CCGCGTTAATGCTAATTGTGATAGGGG
miR-744-5p	TGCGGGGCTAGGGCTAA
miR-423-3p	TAGCTCGGTCTGAGGCCC
miR-23b-5p	GGGTTCCTGGCATGCTGATTT
Let-7c-5p	TGAGGTAGTAGGTTGTATGGTT
miR-362-5p	CGGAATCCTTGGAACCTAGGTGTGAAT

miR-200b-3p	CGCCGTAATACTGCCTGGTAATGATGA
miR-200a-3p	CGCCTAACACTGTCTGGTAACGATGT
U6	CAAGGATGACACGCAAATTCG

1. Li W, He T, Shi R, Song Y, Wang L, Zhang Z, et al. Oligodendrocyte Precursor Cells Transplantation Improves Stroke Recovery via Oligodendrogenesis, Neurite Growth and Synaptogenesis. Aging Dis. 2021; 12: 2096-112.

2. Luo L-L, Li Y-F, Shan H-M, Wang L-P, Yuan F, Ma Y-Y, et al. L-glutamine protects mouse brain from ischemic injury via up-regulating heat shock protein 70. CNS Neurosci Ther. 2019; 25: 1030-41.

3. Ma Y, Jiang L, Wang L, Li Y, Liu Y, Lu W, et al. Endothelial progenitor cell transplantation alleviated ischemic brain injury via inhibiting C3/C3aR pathway in mice. J Cereb Blood Flow Metab. 2020; 40: 2374-86.

4. Mamtilahun M, Jiang L, Song Y, Shi X, Liu C, Jiang Y, et al. Plasma from healthy donors protects blood-brain barrier integrity via FGF21 and improves the recovery in a mouse model of cerebral ischaemia. Stroke Vasc Neurol. 2021; 6: 561-71.

5. Li Z, Song Y, He T, Wen R, Li Y, Chen T, et al. M2 microglial small extracellular vesicles reduce glial scar formation via the miR-124/STAT3 pathway after ischemic stroke in mice. Theranostics. 2021; 11: 1232-48.

6. Deacon RMJ, Rawlins JNP. T-maze alternation in the rodent. Nat Protoc. 2006; 1: 7-12.

7. Wang L, Geng J, Qu M, Yuan F, Wang Y, Pan J, et al. Oligodendrocyte precursor cells transplantation protects blood-brain barrier in a mouse model of brain ischemia via

Wnt/β-catenin signaling. Cell Death Dis. 2020; 11: 9.

8. Mu H-F, Gao X-G, Li S-C, Wei P-J, Zhao Y-F, Zhang W-T, et al. Distinctive functional deficiencies in axonal conduction associated with two forms of cerebral white matter injury. CNS Neurosci Ther. 2019; 25: 1018-29.
